# Chromatin accessibility governs the differential response of cancer and T cells to arginine starvation

**DOI:** 10.1016/j.celrep.2021.109101

**Published:** 2021-05-11

**Authors:** Nicholas T. Crump, Andreas V. Hadjinicolaou, Meng Xia, John Walsby-Tickle, Uzi Gileadi, Ji-Li Chen, Mashiko Setshedi, Lars R. Olsen, I-Jun Lau, Laura Godfrey, Lynn Quek, Zhanru Yu, Erica Ballabio, Mike B. Barnkob, Giorgio Napolitani, Mariolina Salio, Hashem Koohy, Benedikt M. Kessler, Stephen Taylor, Paresh Vyas, James S.O. McCullagh, Thomas A. Milne, Vincenzo Cerundolo

**Affiliations:** 1 MRC Molecular Haematology Unit, MRC Weatherall Institute of Molecular Medicine, NIHR Oxford Biomedical Research Centre Haematology Theme, Radcliffe Department of Medicine, University of Oxford, Oxford OX3 9DS, UK; 2 MRC Human Immunology Unit, MRC Weatherall Institute of Molecular Medicine, University of Oxford, Oxford OX3 9DS, UK; 3 Chemistry Research Laboratory, Department of Chemistry, University of Oxford, Oxford OX1 3TA, UK; 4 Section for Bioinformatics, DTU Health Technology, Technical University of Denmark, Lyngby, Denmark; 5 School of Cancer and Pharmaceutical Sciences, King’s College London, SGDP Centre, Memory Lane, London SE5 8AF, UK; 6 Target Discovery Institute, Centre for Medicines Discovery, Nuffield Department of Medicine, University of Oxford, Oxford OX3 7FZ, UK; 7 MRC WIMM Centre for Computational Biology, MRC Weatherall Institute of Molecular Medicine, Radcliffe Department of Medicine, University of Oxford, Oxford OX3 9DS, UK

**Keywords:** immunosuppression, immunometabolism, cancer metabolism, nutritional stress, metabolic regulation, arginine, T cell chromatin, ASS1, ATF4, H3K27me3

## Abstract

Depleting the microenvironment of important nutrients such as arginine is a key strategy for immune evasion by cancer cells. Many tumors overexpress arginase, but it is unclear how these cancers, but not T cells, tolerate arginine depletion. In this study, we show that tumor cells synthesize arginine from citrulline by upregulating argininosuccinate synthetase 1 (*ASS1*). Under arginine starvation, *ASS1* transcription is induced by ATF4 and CEBPβ binding to an enhancer within *ASS1*. T cells cannot induce *ASS1*, despite the presence of active ATF4 and CEBPβ, as the gene is repressed. Arginine starvation drives global chromatin compaction and repressive histone methylation, which disrupts ATF4/CEBPβ binding and target gene transcription. We find that T cell activation is impaired in arginine-depleted conditions, with significant metabolic perturbation linked to incomplete chromatin remodeling and misregulation of key genes. Our results highlight a T cell behavior mediated by nutritional stress, exploited by cancer cells to enable pathological immune evasion.

## Highlights

• Arginine starvation induces *ASS1* expression in some cancers but not in T cells
• ATF4 binds an internal *ASS1* enhancer to drive expression in cancer but not T cells
• T cell activation is disrupted by arginine starvation, with a loss of reprogramming
• Arginine starvation compacts chromatin in T cells, disrupting ATF4 binding

## Graphical abstract

**Figure:**
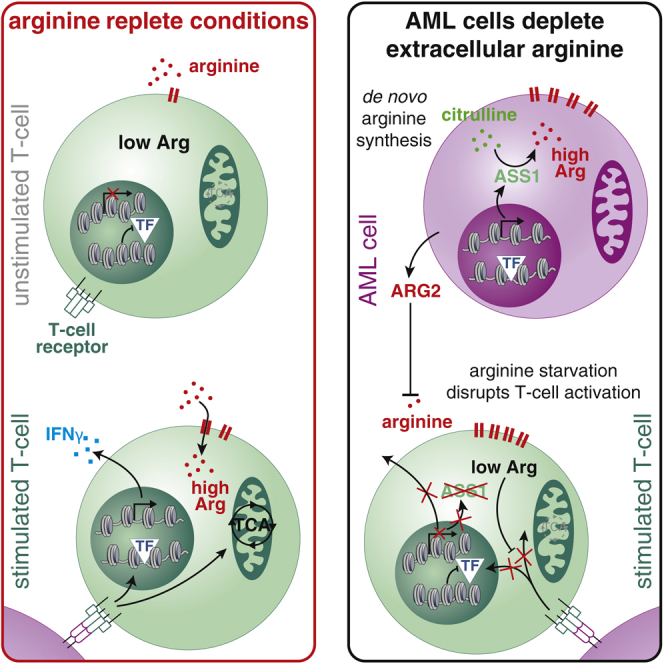

## Teaser

Arginine depletion by many tumors generates an immunosuppressive microenvironment. Crump et al. show that cancer cells can tolerate this by inducing ATF4-dependent upregulation of *ASS1*, allowing *de novo* arginine synthesis. T cells are unable to synthesize arginine as *ASS1* is repressed, disrupting T cell function and the chromatin remodeling associated with activation.

## Introduction

The concept of cancer immunosurveillance has been cemented with the success of immune checkpoint inhibitors (Dunn et al., 2004; Hamid et al., 2013; Hodi et al., 2010; Kim et al., 2007; Topalian et al., 2012; Wolchok et al., 2013). The host immune system can detect and respond to tumors (Gajewski et al., 2013; Hadrup et al., 2013; Schreiber et al., 2011), and infiltration of tumors by T cells predicts a better clinical outcome (Galon et al., 2006). Evasion of the anti-tumor immune response is a hallmark of cancer, and tumors have evolved strategies to achieve this (Hanahan and Weinberg, 2011; Spranger and Gajewski, 2018; Wellenstein and de Visser, 2018). One mechanism is nutritional stress, where tumors exploit the metabolic requirements of T cells and generate an immunosuppressive microenvironment by enzyme-mediated degradation of amino acids (reviewed in Timosenko et al., 2017).

As a semi-essential amino acid, arginine availability is important in determining the immune system’s ability to respond to cancer. Arginase-1 (ARG1) and arginase-2 (ARG2), which convert arginine to ornithine and urea, are expressed or secreted by various tumors such as acute myeloid leukemia (AML), prostate, breast, and neuroblastoma, as well as cancer-associated cells such as fibroblasts, tumor-associated macrophages, and myeloid-derived suppressor cells (MDSCs) (Timosenko et al., 2017). Depletion of arginine is a significant regulator of immune activity in various physiological and pathological circumstances, including pregnancy, the response to infectious agents, autoimmune disease, and cancer, dictating T cell growth and activity (Munder et al., 2006; Rodriguez et al., 2007; Timosenko et al., 2017). Arginine deficiency in the tumor microenvironment impairs T cell responses by CD3ζ downregulation, inhibition of proliferation, and hindrance of cytokine release (Bronte and Zanovello, 2005; Rodriguez et al., 2004; Zea et al., 2004). Conversely, high arginine levels enhance T cell survival and anti-tumor activity (Geiger et al., 2016), and *Arg2* deletion in murine CD8 T cells has a similar effect (Martí i Líndez et al., 2019).

While many cancers are arginine auxotrophs, dependent on extracellular supply (Delage et al., 2010), a significant subset can tolerate low arginine conditions (Mussai et al., 2013; Szlosarek et al., 2007). The inability of T cells to grow in the absence of arginine (Rodriguez et al., 2007; Zea et al., 2004) suggests that a unique pathway must exist in low arginine-tolerant tumors to deal with arginine loss.

Expression of urea cycle enzymes is a potential driver of arginine independence, facilitating *de novo* synthesis. Argininosuccinate synthetase (*ASS1*) expression differs between cancers, being silenced in a number of auxotrophic tumors (Ensor et al., 2002; Fiedler et al., 2015; Kobayashi et al., 2010; Liu et al., 2017; Nicholson et al., 2009; Ohshima et al., 2017; Syed et al., 2013; Szlosarek et al., 2017; Werner et al., 2019) but expressed in other cancers (Delage et al., 2010; Henriet et al., 2017; Rho et al., 2008; Szlosarek et al., 2007; Tsai et al., 2018). Human T cells do not express *ASS1* (Ohno et al., 1992; Sugimura et al., 1990), and chimeric antigen receptor (CAR)-T cells engineered to express *ASS1* show enhanced proliferation and *in vivo* anti-tumor activity (Fultang et al., 2020), indicating that inducing expression is a mechanism to tolerate arginine depletion. DNA methylation represses *ASS1* in a subset of cancers (Miraki-Moud et al., 2015; Nicholson et al., 2009; Syed et al., 2013), but it is unclear what limits expression in T cells.

Here, we show that cells derived from AML and other tumors are able to grow in the absence of extracellular arginine, using the precursor metabolite citrulline for *de novo* synthesis. This is dependent on upregulation of *ASS1*, induced by binding of the transcription factors (TFs) ATF4 and CEBPβ to an intronic enhancer. T cells cannot adequately induce *ASS1*, despite the presence of ATF4 and CEBPβ, as expression is suppressed by a repressive chromatin environment, marked by histone H3 lysine-9/lysine-27 trimethylation (H3K9me3/H3K27me3) and reduced DNA accessibility. Arginine starvation of T cells results in genome-wide chromatin compaction and increased H3K9me3/H3K27me3, disrupting ATF4 and CEBPβ binding at target genes. Finally, we show that the substantial epigenomic, transcriptional, and metabolic reprogramming that normally occurs upon T cell activation is perturbed in the absence of arginine, meaning that arginine-starved T cells retain many of the phenotypic features of unstimulated cells.

## Results

### AML cells but not T cells use citrulline as an alternative arginine source

**Figure 1:**
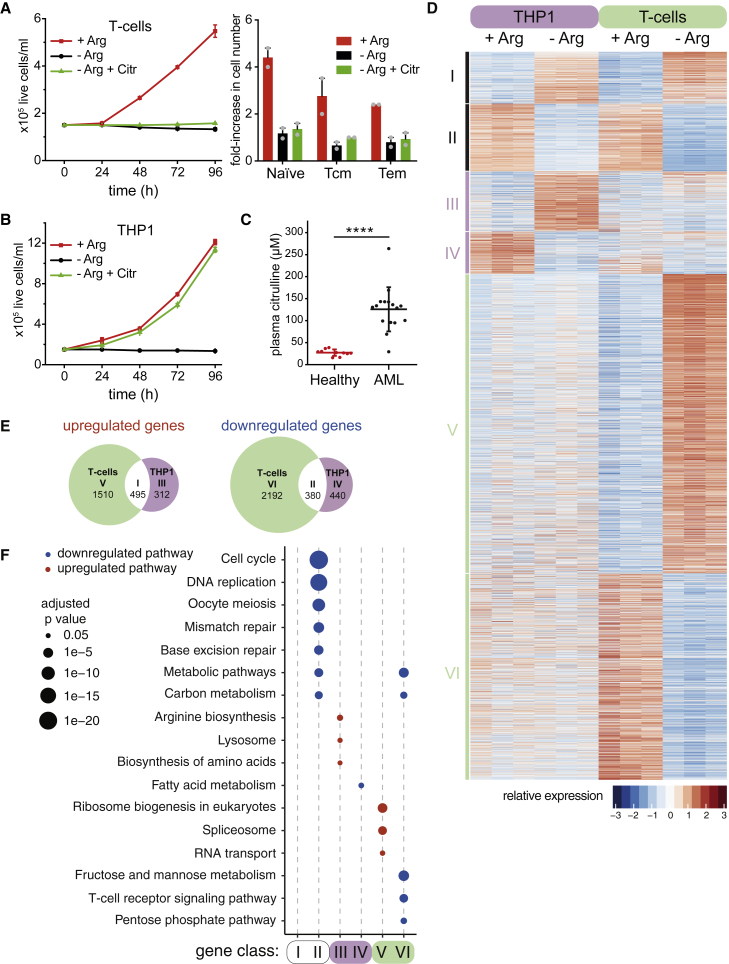
T cells and THP1 cells show differential responses to arginine starvation (A) Left: growth of stimulated CD4^+^ human T cells in complete (+Arg) or arginine-free medium with (−Arg +Citr) or without (−Arg) citrulline. Data are represented as mean ± SD; n = 4. Right: naive, central memory (Tcm), and effector memory (Tem) T cells (see Figure S1D for sort strategy) were stimulated and then incubated in the indicated media for 96 h and counted. Data are fold increase over cell number at 0 h; mean ± SD; n = 2. (B) Growth of THP1 cells in the indicated media. Data are represented as mean ± SD; n = 4. (C) Concentration of citrulline in the blood plasma of healthy (control) and plasma or bone marrow of AML patients. Center bar shows mean ± SD. ^∗∗∗∗^p < 0.0001 (unpaired t test). (D) Microarray analysis of mRNA in THP1 or stimulated T cells incubated in +Arg or −Arg medium for 72 h. Each column represents a replicate. Class assignments (I–VI) for genes are indicated. (E) Overlap of differentially expressed genes in T cells and THP1 cells, with class assignments (I–VI) indicated. (F) Analysis of KEGG pathway enrichment within each class of differentially expressed genes following arginine starvation, shown in (D). Dot size is proportional to significance (Wallenius method). See also Figure S1 and Tables S1 and S2.

To model the response of T cells to arginine restriction, we stimulated primary human CD4^+^ T cells and monitored growth in complete (+Arg, ∼1 mM arginine) or arginine-free (−Arg) RPMI 1640. No proliferation was observed under arginine starvation, and this was not rescued by addition of the arginine precursor citrulline (Figure 1A). Cells responded to stimulation, as CD25, a late T cell activation marker, was upregulated in T cells stimulated in −Arg medium (Figure S1A). However, decreased cell-surface CD3 coreceptor levels and interferon-γ (IFNγ) expression (Munder et al., 2006; Rodriguez et al., 2002; Zea et al., 2004) indicated incomplete activation (Figures S1B and S1C). The inability to use citrulline for growth was a general characteristic of human CD4^+^ T cells, as we saw the same effect with isolated naive, central memory (Tcm), and effector memory (Tem) T cells (Figures 1A, right, and S1D). Low, but non-zero, plasma arginine levels are sometimes observed in AML patients (Mussai et al., 2015; Mussai et al., 2019). In contrast to other work (Werner et al., 2017), we found that low (20 μM) arginine did not restore significant T cell growth in the presence or absence of citrulline (Figures S1E and S1F), suggesting that both low and −Arg levels have immunosuppressive effects.

The reduced serum arginine in some AML patients (Mussai et al., 2015, 2019) suggests that, unlike T cells, AML tumor cells can tolerate this environment. To understand the mechanism underlying this difference, we used the AML cell line THP1. While THP1 cells did not grow in medium lacking arginine, proliferation was rescued by citrulline supplementation (Figure 1B), suggesting its use as an alternative arginine source. This was recapitulated by incubating THP1 cells in +Arg medium treated with recombinant human ARG2 (Figure S1G), mimicking the plasma conditions of AML patients (Mussai et al., 2013). To test citrulline availability *in vivo*, we measured levels in the plasma of AML patients, identifying significant elevation relative to healthy donors (Figure 1C), at concentrations sufficient for growth of arginine-starved THP1 cells *in vitro* (Figure S1H). This argues that citrulline in the plasma of these AML patients allows cancer cells, but not T cells, to synthesize arginine.

### Arginine starvation results in large-scale transcriptional changes

To understand the response to arginine starvation, we analyzed the transcriptional profile of stimulated T cells and THP1 cells incubated in +Arg or −Arg medium (Table S1). We identified 5,445 differentially expressed genes in response to arginine starvation (Figure 1D), of which 875 responded similarly in THP1 and T cells and 4,454 responded only in one cell type (Figures 1E and S1I). A much larger number were differentially expressed in T cells (4,577) than in THP1 cells (1,627), indicating a stronger response to loss of arginine. 5,329 genes fell into six classes: upregulated or downregulated in both cell types (I and II, respectively; Figures 1D and 1E), THP1 cells only (III and IV), or T cells only (V and VI). Cell cycle and DNA replication-related genes were downregulated in both THP1 and T cells (class II; Figure 1F), consistent with growth inhibition (Figures 1A and 1B). T cell receptor signaling pathway genes were specifically downregulated in T cells (class VI; Figures 1F and S1J; Table S2), matching the loss of activation markers in −Arg cells (Figures S1B and S1C).

**Figure 2:**
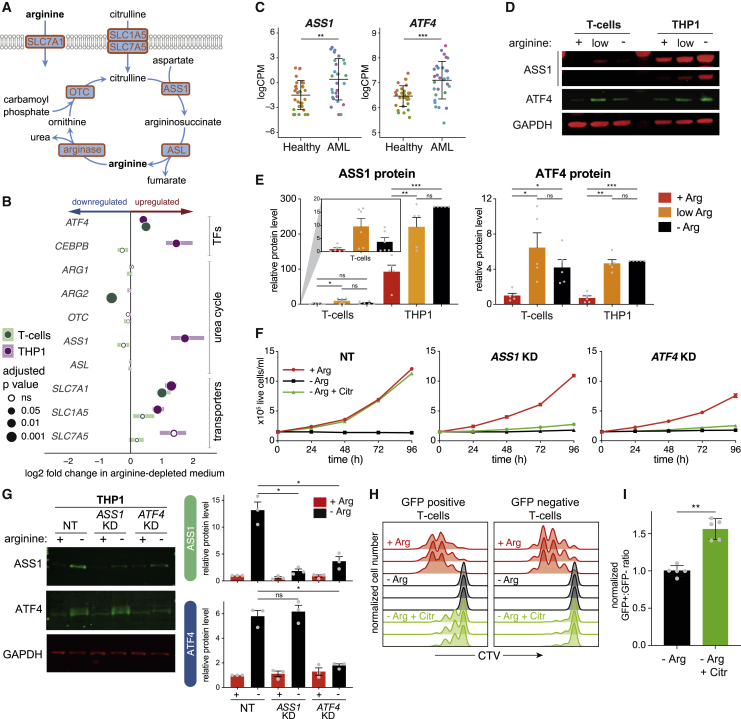
ATF4-induced *ASS1* upregulation facilitates citrulline-dependent growth of THP1 cells (A) Key proteins in arginine uptake and biosynthesis. (B) Forest plot showing changes in gene expression, based on microarray analysis (see Figure 1D). Horizontal bars show interquartile range. (C) *ASS1* and *ATF4* expression in primary AML blasts or non-transformed monocytic and myelocytic cells from healthy donors (Quek et al., 2016). Samples are colored by donor. Bars show mean ± SD. ^∗∗^p < 0.01, ^∗∗∗^p < 0.001 (Mann-Whitney test). (D) Western blot for ASS1 and ATF4 in stimulated T cells and THP1 cells incubated for 72 h in complete medium (+), medium containing 20 μM arginine (low), or lacking arginine (−). Two exposures of the ASS1 blot are shown for clarity. Representative of five replicates. (E) Quantification of (D), normalized to GAPDH, relative to +Arg T cells. Expression in T cells is shown on a smaller scale for clarity. Data are represented as mean ± SEM; n = 5. ^∗^p < 0.05, ^∗∗^p < 0.01, ^∗∗∗^p < 0.001 (Dunnett’s multiple comparison test). ns, not significant. (F) Growth of control (NT) THP1 cells or following KD of *ASS1* or *ATF4* in the indicated media. Data are represented as mean ± SD; n = 4. (G) Representative western blot for ASS1 and ATF4 in control (NT) THP1 cells or following KD of *ASS1* or *ATF4* in the presence (+) and absence (−) of arginine for 72 h. Right: quantification, normalized to GAPDH, relative to +Arg NT cells. Data are represented as mean ± SEM; n = 3. ^∗^p < 0.05 (Dunnett’s multiple comparison test). (H) CTV-labeled CD8^+^ T cells were transduced with a *GFP*-*ASS1* coexpression plasmid. Cells were analyzed for GFP and CTV levels after 96 h in the indicated media. Three technical replicates are shown. (I) Proportion of GFP-positive cells from the analysis in (H), normalized to the −Arg ratio. Data are represented as mean ± SD; n = 5 from two donors. ^∗∗^p < 0.01 (paired t test). See also Figure S2.

Intracellular availability of arginine depends on extracellular uptake by amino acid transporters and/or *de novo* synthesis from citrulline and other urea cycle precursors (Figure 2A), suggesting that the discrepancy between tumor and T cell behavior may reflect differences in these processes. The arginine transporter gene *SLC7A1* (CAT1) (Closs et al., 2004) was upregulated in both THP1 and T cells (Figures 2B, S2A, and S2B), arguing that the difference is not due to an inability to import arginine. SLC1A5 (ASCT2) and SLC7A5 (LAT1) are neutral amino acid transporters, with the latter implicated in citrulline uptake (Werner et al., 2017). To test this, we treated THP1 cells with the SLC1A5 and SLC7A5 inhibitors GPNA and BCH, respectively (Christensen, 1990; Esslinger et al., 2005; Kim et al., 2002). Each produced a moderate reduction in citrulline-dependent growth, enhanced by co-treatment, suggesting that both transporters are important for citrulline uptake (Figure S2C). Mass spectrometric analysis confirmed that THP1 cells could take up extracellular citrulline and generate intracellular arginine *de novo* (Figure S2D). *SLC7A5* tended to be more strongly upregulated in THP1 cells compared to T cells upon arginine starvation (Figures 2B and S2A). However, all three transporters were upregulated upon T cell activation (Figure S2B), and expression levels were comparable between arginine-starved THP1 and T cells (Figure S2A), suggesting that transporter availability may not explain the differential response.

### ASS1 expression drives the tolerance of THP1 cells to arginine starvation

THP1 cells, but not T cells, upregulated genes involved in arginine biosynthesis (Figure 1F), which may allow starvation tolerance. Of the urea cycle genes, *ASS1*, which regulates the rate-limiting step of the pathway (Haines et al., 2011), showed significantly increased RNA levels in THP1 cells, but not T cells, following arginine starvation (Figures 2B and S2E), suggesting a key role in promoting growth in the absence of arginine. AML patient RNA sequencing (RNA-seq) data (Quek et al., 2016) also revealed elevated *ASS1* expression (Figure 2C). Levels of *ASS1* protein were also increased in arginine-starved THP1 cells, but not T cells, where basal levels were already very low (Figures 2D and 2E). Similar to previous reports (Werner et al., 2017), we observed a weak induction of *ASS1* in bulk T cells, as well as isolated naive, Tcm, and Tem cells, under low arginine conditions (Figures 2D, 2E, S2E, and S2F).

*ASS1* knockdown (KD) strongly disrupted the ability of THP1 cells to use citrulline for growth (Figures 2F and 2G), demonstrating its key role in the arginine starvation response. As the inability to express *ASS1* appears to explain the lack of citrulline-driven T cell growth, we asked whether it could be rescued by exogenous *ASS1* expression. Indeed, transduction of stimulated CD8^+^ T cells with an *ASS1*-*GFP* plasmid enabled the growth of GFP-positive, but not untransduced GFP-negative, cells using citrulline as an alternative arginine source (Figures 2H and 2I). This is consistent with recent work showing that *ASS1*-expressing CAR-T cells are more effective at targeting tumor cells *in vivo* (Fultang et al., 2020), suggesting a potential therapeutic strategy to rescue T cell growth in arginine-depleted patients.

### *ASS1* transcription is induced by an ATF4-bound enhancer

Analysis of TF binding sites at genes upregulated in THP1 cells (class III; Figure 1D) revealed enrichment for ATF4 motifs (Figure S2G). Both *ATF4* and its binding partner *CEBPB* (Ameri and Harris, 2008; Vinson et al., 1993) were upregulated in arginine-starved THP1 cells (Figure 2B), and *ATF4* is upregulated in primary blasts from *ASS1*-expressing AML patients (Figure 2C). ATF4 protein was strongly induced in both THP1 and T cells (Figures 2D and 2E) in response to arginine restriction (Harding et al., 2000; Lu et al., 2004; Vattem and Wek, 2004). *ATF4* KD abrogated both *ASS1* expression and citrulline-dependent growth in THP1 cells (Figures 2F and 2G), implicating ATF4 in *ASS1* induction. Importantly, the observation that ATF4 was also upregulated in T cells, without inducing *ASS1* (Figures 2D and 2E), suggests that its ability to activate target genes is restricted in T cells.

**Figure 3:**
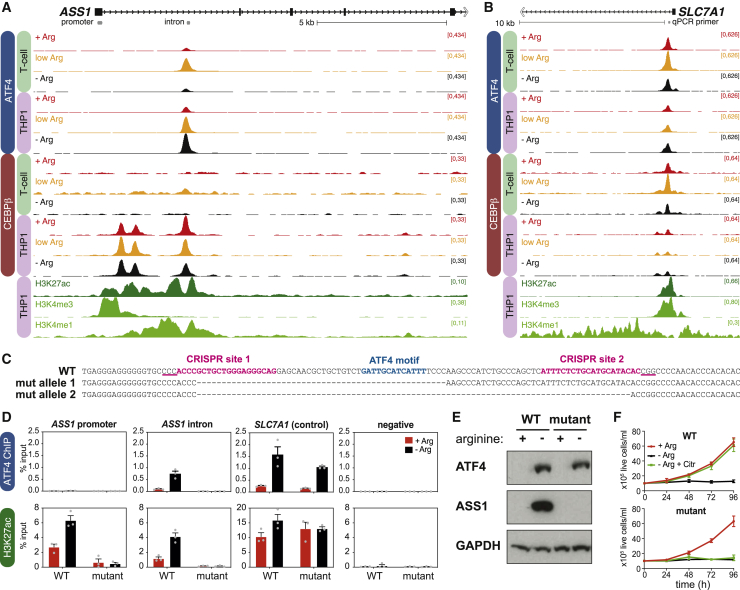
ATF4 activates *ASS1* transcription via an intronic enhancer (A) Reference-normalized ChIP-seq for ATF4 and CEBPβ at *ASS1* in stimulated T cells and THP1 cells incubated in the indicated media for 72 h, and ChIP-seq for H3K4me3, H3K27ac, and H3K4me1 in THP1 cells in +Arg medium (Godfrey et al., 2019). Gray bars show qPCR primer locations. (B) Reference-normalized ChIP-seq for ATF4 and CEBPβ at *SLC7A1*, as in (A). (C) Sequences of the enhancer region in parental (wild type [WT]) and mutant THP1 cells. PAM sequences are underlined. (D) ChIP-qPCR for ATF4 and H3K27ac in WT and mutant THP1 cells, incubated in +Arg or −Arg medium for 72 h. Data are represented as mean ± SEM; n = 3. (E) Western blot for ASS1 and ATF4 in WT and mutant THP1 cells, incubated in +Arg or −Arg medium for 72 h. Representative of three replicates. (F) Growth of WT and mutant THP1 cells, incubated in the indicated media. Data are represented as mean ± SD; n = 3. See also Figure S3.

To test whether ATF4 directly activates *ASS1*, we performed reference-normalized chromatin immunoprecipitation sequencing (ChIP-seq) (Orlando et al., 2014) for ATF4 and CEBPβ in THP1 and T cells in +Arg, low arginine, or −Arg medium. ATF4 and CEBPβ bound *ASS1* within the first intron in THP1 cells (Figure 3A), associated with ATF4/CEBPβ motifs (Figure S3A). In −Arg T cells, where *ASS1* is not expressed, only a low level of ATF4 and CEBPβ binding was observed (Figures 3A and S3B). In contrast, both ATF4 and CEBPβ were bound at the promoter of *SLC7A1* at comparable levels in T cells and THP1 cells (Figures 3B and S3B). This is consistent with the induction of *SLC7A1*, but not *ASS1*, in arginine-starved T cells (Figure 2B) and suggests that an extra layer of control regulates ATF4 binding at *ASS1*.

The ATF4 binding site at *ASS1* is marked by H3K4me1 and H3K27 acetylation (H3K27ac; Figure 3A) in THP1 cells, suggesting that it may be an enhancer for *ASS1*. CRISPR-Cas9-mediated deletion of this region in THP1 cells (Figures 3C and S3C) disrupted ATF4 and CEBPβ binding specifically at the *ASS1* enhancer and strongly reduced H3K27ac at both the promoter and intron of *ASS1*, but not at *SLC7A1* (Figures 3D and S3D). Mutant clones could not induce *ASS1* expression (Figures 3E, S3E, and S3F) or use citrulline in place of arginine for growth (Figures 3F and S3G). Taken with the results of *ATF4* KD (Figure 2G), this strongly argues that the ATF4/CEBPβ binding site within *ASS1* acts as an enhancer for the gene.

### *ASS1* is repressed in T cells

In T cells under low arginine conditions, ATF4 binds to the *ASS1* enhancer but CEBPβ binding is lower than in THP1 cells (Figures 3A and S3B), and this does not strongly activate *ASS1* expression (Figures 2D and 2E). We asked what might regulate *ASS1* expression and ATF4/CEBPβ binding in T cells. *ASS1* is commonly repressed by DNA methylation in arginine auxotrophic tumors (Miraki-Moud et al., 2015; Nicholson et al., 2009; Syed et al., 2013). However, the *ASS1* promoter and enhancer were either almost completely unmethylated in both THP1 and T cells, or they did not show a clear difference between cell types or conditions (Figure S4A). Thus, DNA methylation is unlikely to regulate ATF4/CEBPβ binding or *ASS1* expression in T cells.

**Figure 4:**
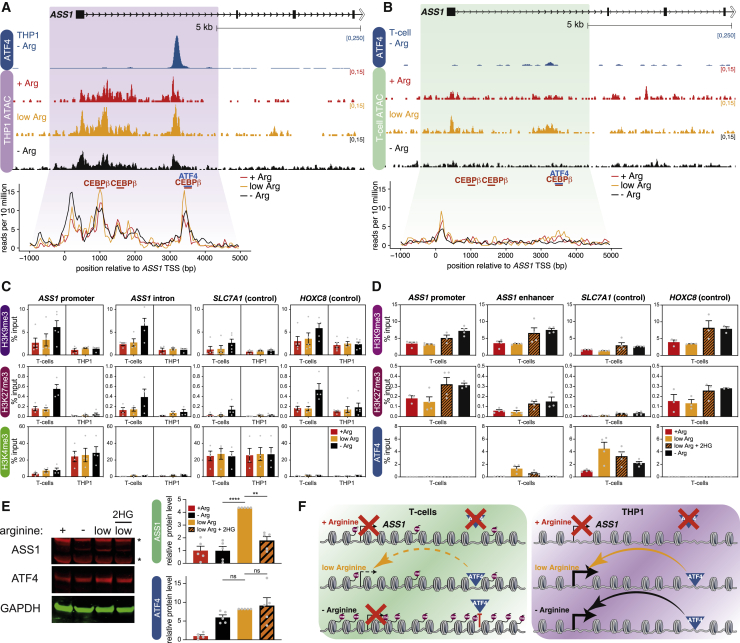
*ASS1* is repressed in T cells (A) ATAC-seq at *ASS1* in THP1 cells incubated in the indicated media for 72 h. ATF4 ChIP-seq from −Arg cells is shown for comparison. Bottom: overlay of ATAC-seq traces at the highlighted region of *ASS1*, mean of three replicates. (B) ATAC-seq at *ASS1* in stimulated T cells, as in (A). (C) ChIP-qPCR for H3K9me3, H3K27me3, and H3K4me3 in stimulated T cells and THP1 cells incubated in the indicated media for 72 h. Data are represented as mean ± SEM; n = 4. (D) ChIP-qPCR for H3K9me3, H3K27me3, and ATF4 in stimulated T cells incubated for 72 h in complete medium (+Arg), medium containing 20 μM arginine, without (low Arg) or with (low Arg + 2HG) addition of 500 μM 2HG, or lacking arginine (−Arg). Data are represented as mean ± SEM; n = 4. (E) Representative western blot for ASS1 and ATF4 in stimulated T cells incubated in the indicated media for 72 h. Non-specific bands are indicated by an asterisk. Right: quantification, normalized to GAPDH, relative to +Arg. Data are represented as mean ± SEM; n = 5. ^∗∗^p < 0.01, ^∗∗∗∗^p < 0.0001 (Dunnett’s multiple comparison test). (F) Model for *ASS1* regulation in T cells and THP1 cells in response to arginine depletion. In THP1 cells accessibility at *ASS1* allows ATF4 binding under low and −Arg conditions, inducing *ASS1* expression. In T cells, ATF4 binding and *ASS1* expression are regulated by two competing processes: ATF4 is active under low or −Arg conditions, but the *ASS1* promoter is repressed. Under −Arg, elevated H3K9me3/H3K27me3 and reduced accessibility at *ASS1* block ATF4 binding. See also Figure S4.

We used an assay for transposase-accessible chromatin with high-throughput sequencing (ATAC-seq) to ask whether the differences between THP1 and T cells at *ASS1* correlated with chromatin accessibility. THP1 cells displayed regions of accessibility at both the promoter and enhancer (Figure 4A). The peaks are present in cells in +Arg medium where there is relatively weak ATF4 binding and *ASS1* expression, suggesting that accessibility precedes TF binding and transcription. In T cells, accessibility at the *ASS1* promoter and enhancer is low under all three treatments (+Arg, low Arg, and −Arg; Figure 4B), which may explain the weak expression under low arginine conditions, when ATF4 is bound (Figure 3A). Both promoter and enhancer show slight compaction under arginine starvation (Figure 4B), suggesting that reduced accessibility may block ATF4 binding. In contrast, the promoter of *SLC7A1* remained accessible under all three arginine conditions in THP1 and T cells (Figure S4B), consistent with ATF4/CEBPβ binding inducing transcription in both cell types.

To understand what drives the differences in *ASS1* expression, chromatin accessibility, and TF binding, we looked for the presence of repressive histone modifications. Levels of H3K9me3 and H3K27me3 at the promoter and enhancer of *ASS1* were noticeably higher in T cells than THP1, matching levels at the repressed *HOXC8* gene, and depleted at *SLC7A1* (Figure 4C). Conversely, H3K4me3, a mark of active and poised genes, was considerably lower at the *ASS1* promoter in T cells compared to THP1 (Figure 4C). This suggests that elevated H3K9me3/H3K27me3 at *ASS1* may explain the inability of ATF4/CEBPβ to strongly induce expression in T cells.

In addition to the high basal levels of H3K9me3 and H3K27me3 at *ASS1* in T cells, arginine starvation resulted in striking increases in methylation (Figure 4C). We asked whether this had a causative role in restricting ATF4 binding and *ASS1* expression by artificially inducing methylation with the demethylase inhibitor 2-hydroxyglutarate (2HG) (Chowdhury et al., 2011; Xu et al., 2011). Treating T cells under low arginine conditions with 2HG produced a clear increase in H3K9me3 and H3K27me3, comparable to the effect of arginine starvation (Figure 4D). Strikingly, 2HG treatment disrupted ATF4 binding at *ASS1*, mimicking −Arg conditions (Figure 4D). *ASS1* expression was downregulated to near arginine-starvation levels (Figures 4E and S4C). Taken together, this suggests a model where ATF4 binding in T cells under low arginine conditions cannot strongly upregulate *ASS1* expression owing to high basal levels of H3K9me3/H3K27me3 and poor accessibility. In arginine-starved T cells, elevated levels of repressive histone modifications disrupt ATF4 binding at *ASS1* and silence transcription (Figure 4F).

2HG is upregulated in CD8^+^ T cells under hypoxic conditions, increasing H3K27me3 (Tyrakis et al., 2016). We asked whether arginine starvation produced elevated H3K9me3/H3K27me3 by a similar mechanism. However, we saw no significant difference in 2HG levels between T cells stimulated in +Arg or −Arg medium (Figure S4D), indicating that distinct processes regulate H3K9me3/H3K27me3.

### *ASS1* induction is associated with tolerance to arginine starvation in multiple tumor cell lines

**Figure 5:**
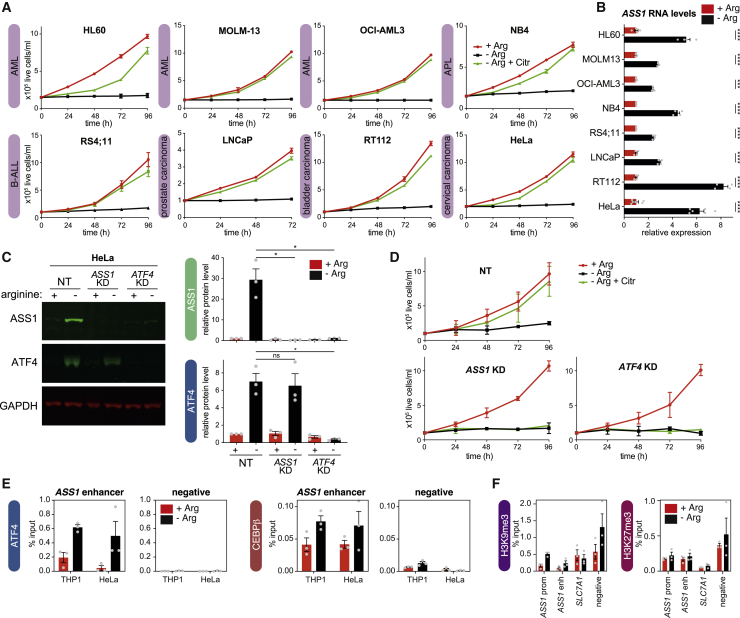
*ASS1* upregulation is a common tumor response to arginine starvation (A) Growth of tumor cell lines in the indicated media. AML, acute myeloid leukemia; APL, acute promyelocytic leukemia; ALL, acute lymphoblastic leukemia. Data are represented as mean ± SD; n = 4. (B) qRT-PCR for *ASS1* in the indicated cell lines, cultured in +Arg and −Arg media. Data are normalized to *GAPDH*, relative to +Arg in each cell line, represented as mean ± SD; n = 4 or 6 (HeLa). ^∗∗∗^p < 0.001, ^∗∗∗∗^p < 0.0001 (Šidák’s multiple comparison test). (C) Representative western blot for ASS1 and ATF4 in control (NT) HeLa cells or following KD of *ASS1* or *ATF4* in the indicated media for 72 h. (Right) Quantification, normalized to GAPDH, relative to +Arg NT cells. Data are represented as mean ± SEM; n = 3. ^∗^p < 0.05 (Dunnett’s multiple comparison test). (D) Growth of control (NT) HeLa cells or following KD of *ASS1* or *ATF4*, incubated in the indicated media. Data are represented as mean ± SD; n = 3. (E) ChIP-qPCR for ATF4 and CEBPβ levels in THP1 and HeLa cells incubated in the indicated media for 72 h. Data are represented as mean ± SEM; n = 3. (F) ChIP-qPCR for H3K9me3 and H3K27me3 in HeLa cells incubated in the indicated media for 72 h. Data are represented as mean ± SEM; n = 3. See also Figure S5.

We asked whether ATF4-dependent *ASS1* induction was unique to THP1 AML cells, or extended to other tumors, using cancer cell lines established from liquid and solid tumors, including AML (HL60, MOLM-13, and OCI-AML3), acute promyelocytic leukemia (NB4), acute lymphocytic leukemia (RS4;11), and prostate (LNCaP), bladder (RT112), and cervical (HeLa) carcinomas. These lines all proliferated using citrulline in place of arginine (Figure 5A), and they upregulated *ASS1* in −Arg medium (Figure 5B). Thus, upregulation of *ASS1* for *de novo* arginine synthesis may allow multiple cancer types to proliferate under arginine starvation.

We further investigated this in HeLa cells, where *ASS1* and *ATF4* KD each disrupted citrulline-dependent proliferation (Figures 5C and 5D). Consistent with this, both ATF4 and CEBPβ bound to the *ASS1* enhancer in −Arg HeLa cells (Figure 5E). Surprisingly, ATAC-seq revealed no peak of chromatin accessibility at the enhancer (Figure S5A), although it was marked with H3K27ac (Figure S5A) and depleted for repressive histone methylation (Figure 5F). The promoter was clearly accessible (Figure S5A), consistent with strong inducible expression of *ASS1* (Figure S5B). Taken together, these data indicate that the ATF4-driven induction of *ASS1* in response to arginine starvation is not unique to AML cells.

### Repressive chromatin restricts ATF4/CEBPβ binding genome-wide in arginine-starved T cells

**Figure 6:**
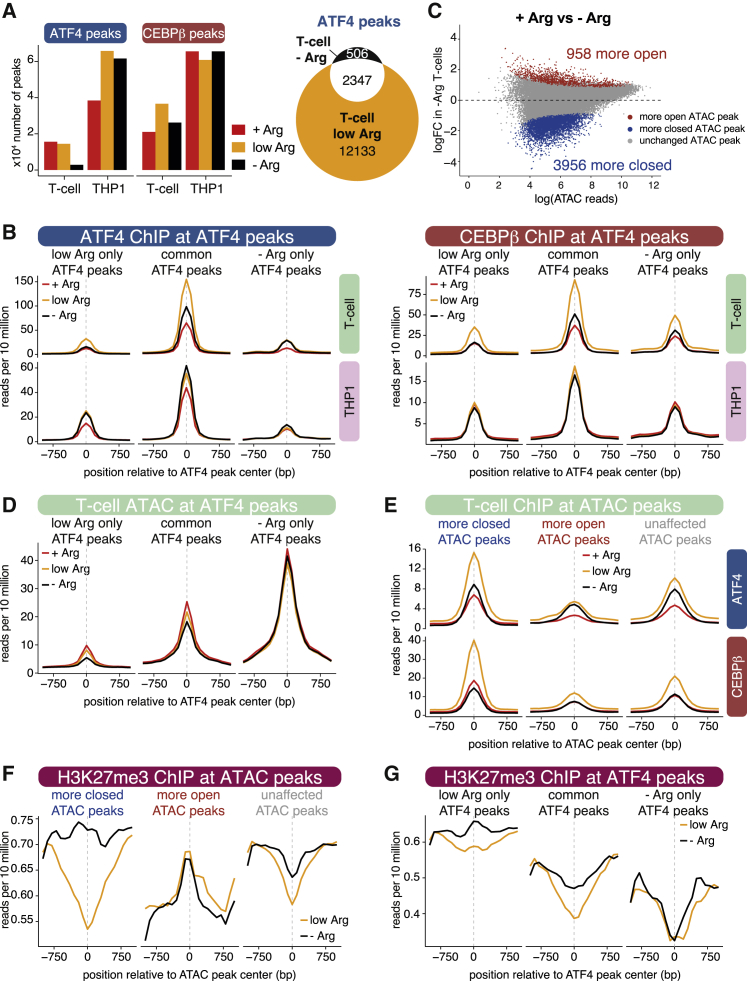
Arginine-starved T cells show reduced ATF4/CEBPβ binding and chromatin accessibility (A) Left: number of ATF4 and CEBPβ peaks identified in ChIP-seq from stimulated T cells and THP1 cells incubated in the indicated media for 72 h. Right: overlap of ATF4 ChIP-seq peaks identified in T cells in low Arg or −Arg conditions. (B) Reference-normalized ATF4 (left) and CEBPβ (right) ChIP-seq levels at ATF4 peaks from T cells and THP1 cells incubated in the indicated media (colored lines). Mean level is displayed for T cell ATF4 peaks found only under low Arg conditions, only under arginine starvation, or under both conditions (common), as in (A). (C) Differential chromatin accessibility between stimulated T cells incubated in +Arg and −Arg medium. Red and blue dots indicate significantly increased and decreased ATAC peaks under −Arg; false discovery rate (FDR) < 0.05. (D) Chromatin accessibility (ATAC-seq) at T cell ATF4 peaks, as in (B). (E) Reference-normalized ATF4 and CEBPβ ChIP-seq levels at ATAC peaks from T cells incubated in the indicated media. Mean level is displayed for peaks that show reduced accessibility (more closed), increased accessibility (more open), or no change (unaffected) in arginine-starved T cells, as in (C). (F) Reference-normalized H3K27me3 ChIP-seq levels at T cell ATAC peaks, as in (E). (G) Reference-normalized H3K27me3 ChIP-seq levels at T cell ATF4 peaks, as in (B). See also Figure S6.

Having demonstrated that arginine starvation of T cells increases repressive histone methylation and disrupts ATF4 binding at *ASS1*, we asked whether this was a more global behavior. We used reference-normalized ATF4 and CEBPβ ChIP-seq to compare binding profiles genome-wide under different arginine conditions. The number of ATF4 and CEBPβ binding sites was markedly higher in THP1 cells than in T cells, indicating that T cells generally show a more restrictive binding environment (Figure 6A). Strikingly, ATF4 binding was much less common in −Arg T cells, with 5-fold fewer peaks than in low arginine T cells (Figure 6A, 2,853 versus 14,480). However, this effect was not observed in THP1 cells, with the number of peaks broadly comparable under each condition. ATF4 binding was much weaker in +Arg T cells (Figure S6A), consistent with the low amount of protein (Figures 2D and 2E). ATF4 protein levels are broadly comparable under low arginine and −Arg T cells (Figures 2D and 2E), so the reduced peak count (Figure 6A) argues that arginine starvation restricts ATF4 binding in T cells but not in THP1 cells.

To investigate the difference in TF binding under arginine restriction, we grouped ATF4 peaks (Figure 6A) based on whether they were present in T cells only under low arginine or −Arg, or under both conditions (common), and analyzed the average peak height (amount of TF bound) in each group. ATF4 and CEBPβ binding was much stronger in low arginine than −Arg T cells at both common and low Arg-only peaks (Figure 6B, upper), matching the stronger binding observed at *ASS1* (Figure 3A). This was not true of THP1 cells, where ATF4/CEBPβ binding was comparable under each condition (Figure 6B, lower). Taken alongside the reduced number of ATF4 peaks (Figure 6A), this provides further evidence that arginine starvation restricts ATF4 and CEBPβ binding at a subset of sites in T cells but not in THP1 cells.

We hypothesized that the decreases in TF binding may be associated with reduced chromatin accessibility, implying genome-wide differences in chromatin structure under arginine starvation. Comparison of ATAC-seq from T cells stimulated in +Arg and −Arg medium revealed significant differences, with a 4-fold bias toward chromatin compaction in −Arg (Figure 6C). To determine whether reduced accessibility correlated with loss of ATF4/CEBPβ binding, we analyzed ATAC-seq levels at ATF4 binding sites (Figure 6D). Accessibility was much lower at sites bound by ATF4 only in low arginine T cells, compared to sites where binding was maintained (common peaks; Figure 6D). This may explain why binding only occurs under low Arg conditions, as even a small increase in compaction could make them inaccessible. Indeed, accessibility was reduced at these peaks in −Arg T cells, correlating with the loss of ATF4 binding (Figure 6D, orange versus black lines). Reduced accessibility also occurred at common ATF4 peaks, but levels remained higher than at low Arg only peaks (Figure 6D). Surprisingly, peaks bound only under −Arg conditions showed a much higher level of accessibility compared to the other two peak sets. This is likely attributable to the fact that many of these peaks are at promoters (Figure S6B), which are typically much more accessible.

Similar results were obtained for the reciprocal analysis: ATAC peaks with decreased accessibility in −Arg T cells (more closed) showed a strong reduction in ATF4 and CEBPβ binding, with a much smaller decrease in binding at unaffected and more open ATAC peaks (Figure 6E). Overall, these results suggest that a subset of loci are bound by ATF4 in low arginine conditions, but these sites become compacted and cannot support binding under starvation.

We used reference-normalized H3K27me3 ChIP-seq to determine whether decreased chromatin accessibility under arginine starvation was associated with increased repressive histone methylation, which could explain reduced ATF4/CEBPβ binding. Strikingly, regions of decreased accessibility (more closed ATAC peaks) saw elevated H3K27me3 under arginine starvation, but unaffected and increased ATAC peaks showed little or no change (Figure 6F). ATF4 peaks that are only present in low Arg conditions displayed increased H3K27me3 levels upon starvation, while −Arg-only peaks showed no change in H3K27me3 (Figure 6G). Thus, there is a correlation between increased H3K27me3, decreased accessibility, and decreased ATF4 binding, arguing that chromatin changes caused by arginine starvation may drive a distinct reprogramming of TF binding and gene expression.

### Arginine starvation disrupts chromatin reorganization associated with T cell activation

**Figure 7:**
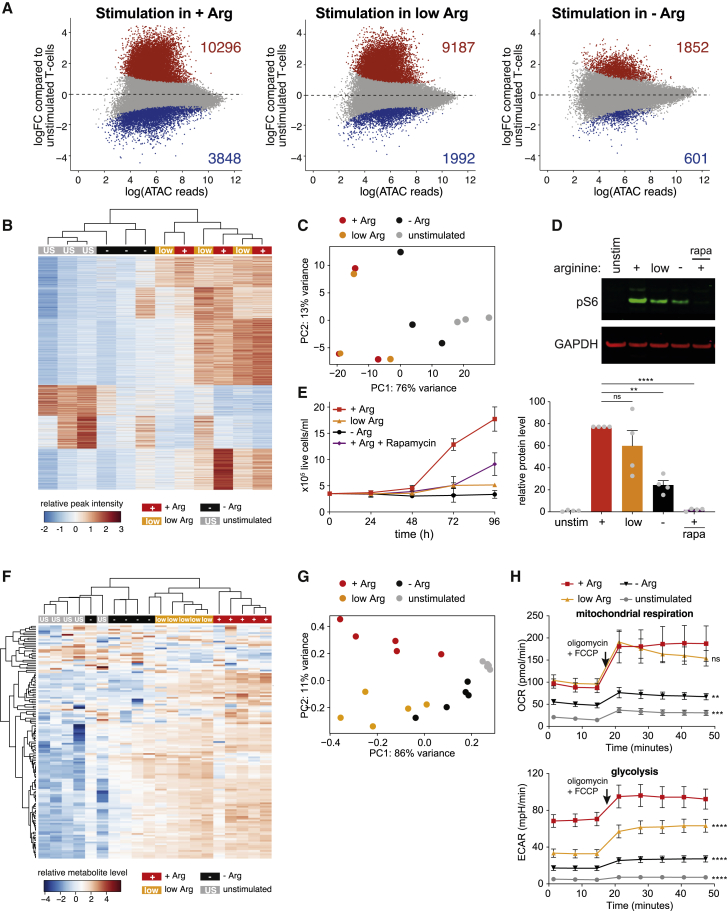
Arginine starvation disrupts chromatin and metabolic reprogramming of T cells (A) Differential chromatin accessibility in T cells upon stimulation in the indicated media for 72 h. Red and blue dots indicate significantly increased and decreased ATAC peaks upon stimulation; FDR < 0.05. (B) K-means clustering (k = 5) of differential ATAC peaks following stimulation in +Arg medium (see A, left), using ATAC-seq from unstimulated T cells (US) and T cells stimulated in the indicated media for 72 h. Each column is a sample, with each row an ATAC peak. (C) Principal component analysis of ATAC-seq data in (B). Each dot represents a sample; n = 3. PC, principal component. (D) Representative western blot of S6 phosphorylation (Ser235/Ser236) in unstimulated T cells and T cells stimulated for 24 h in +Arg, low Arg, or −Arg medium, or +Arg medium in the presence of 20 nM rapamycin (rapa). Bottom: quantification, normalized to GAPDH, relative to unstimulated T cells. Data are represented as mean ± SEM; n = 4. ^∗∗^p < 0.01, ^∗∗∗∗^p < 0.0001 (Dunnett’s multiple comparison test). (E) Growth of T cells stimulated in the indicated media. Data are represented as mean ± SD; n = 5. (F) Hierarchical clustering analysis of metabolite levels from unstimulated T cells (US) and T cells stimulated in the indicated media for 72 h. Each column is a sample, and each row is a metabolite. (G) Principal component analysis of metabolite data in (F). Each dot represents one sample; n = 5. (H) Seahorse analysis of unstimulated T cells and T cells stimulated in the indicated media for 72 h. Data are the mean of four donors ± SEM. ^∗∗^p < 0.01, ^∗∗∗^p < 0.001, ^∗∗∗∗^p < 0.0001, comparisons made with +Arg across all time points (Tukey’s multiple comparison test). See also Figure S7 and Table S3.

A number of studies have linked changes in T cell behavior to reorganization of chromatin structure (Bevington et al., 2016; Gray et al., 2017; Philip et al., 2017; Sen et al., 2016). We asked whether the differences in accessibility we observed upon arginine starvation were related to the inability of the T cells to fully activate (Figures S1B and S1C). T cell stimulation in +Arg medium produced widespread chromatin remodeling, with increased accessibility at most differential peaks (Figure 7A, left). Genes with increased accessibility were enriched in multiple signaling pathways, including T cell receptor signaling (Figure S7A), arguing that remodeling may play an important role in activation.

In contrast to these extensive changes, the effect of stimulation in the absence of arginine was much reduced (Figure 7A, right). The number of differential peaks was more than 5-fold lower (Figure 7A, 2,453 versus 14,144), with a greater similarity between unstimulated and −Arg T cells (Figure S7B). K-means clustering of accessibility at the peaks that respond to stimulation in +Arg medium (Figure 7A, left) grouped unstimulated and −Arg cells separately from T cells stimulated in low arginine and +Arg medium (Figure 7B). Within this group, unstimulated and −Arg T cells clustered separately. Principal component analysis revealed that -Arg T cells have a signature intermediate between unstimulated and +Arg T cells (Figure 7C). This argues that T cells stimulated under arginine starvation are unable to complete the transition to a more open chromatin state, and they do not fully activate.

One of the pathways induced upon T cell activation is mTORC1 signaling (Powell et al., 2012), and mTORC1 activity is dependent on arginine (Bar-Peled and Sabatini, 2014; Chantranupong et al., 2016). Ribosomal protein S6 phosphorylation (downstream of mTORC1) was dramatically reduced under arginine starvation (Figure 7D). Treatment with the mTORC1 inhibitor rapamycin delayed proliferation of stimulated T cells (Figure 7E), although the effect was less pronounced than with arginine starvation. By ATAC-seq, T cells stimulated in the presence of rapamycin clustered more closely to activated T cells, away from unstimulated/−Arg T cells (Figure S7C). Thus, the inability to remodel chromatin in the absence of arginine is not attributable solely to the loss of mTORC1 signaling, suggesting a role for other processes.

### Arginine starvation disrupts metabolic processes

T cell activation is associated with wide-scale metabolic reprogramming, with a switch from solely oxidative phosphorylation to the glycolytic, pentose phosphate, and glutaminolytic pathways (Carr et al., 2010; Chang et al., 2013; Frauwirth et al., 2002; Gubser et al., 2013; Wang et al., 2011). Given the increased metabolic activity of T cells supplemented with arginine (Geiger et al., 2016), we asked what effect arginine starvation had.

We measured the intracellular levels of 145 metabolites, focusing on amino acids and highly polar/ionic compounds (Table S3). Hierarchical clustering grouped unstimulated and −Arg-stimulated T cells (with one exception), separate from T cells stimulated under low or +Arg conditions (Figure 7F). Within this grouping, unstimulated and −Arg T cells tended to cluster separately, suggesting that stimulation under arginine starvation induces metabolic changes, although not to the same extent as in +Arg medium. As with ATAC-seq (Figure 7C), principal component analysis revealed an intermediate metabolite profile for −Arg T cells between unstimulated and +Arg/low Arg T cells, consistent with partial activation (Figure 7G).

In common with other amino acids, arginine levels were low in unstimulated T cells (Figure S7D). Most amino acids showed similar levels in all stimulated conditions, suggesting that the activation-induced increases are not dependent on arginine. We observed a reduction of urea cycle intermediates in unstimulated and −Arg cells (Figure S7E), indicating downregulation of arginine metabolism. Previous work has reported upregulation of *ARG2* upon T cell activation, alongside reduced intracellular arginine levels (Geiger et al., 2016). However, in our hands *ARG2* induction did not lead to arginine depletion (Figures S7D and S7F). Arginine starvation disrupted *ARG2* upregulation (Figure S7F), which may be to conserve intracellular arginine, or as a consequence of incomplete activation. We observed decreases in most nucleotides in both −Arg and unstimulated T cells (Figure S7D). Nucleotide synthesis is upregulated in activated T cells (Geiger et al., 2016), suggesting that arginine starvation may block this process, consistent with impaired T cell activation, potentially by limiting energy availability and/or DNA/RNA synthesis.

Many tricarboxylic acid (TCA) cycle intermediates were strongly depleted in both unstimulated and −Arg T cells (Figure S7G), suggesting a failure of arginine-starved T cells to upregulate the cycle and/or net cataplerotic efflux from the mitochondria matrix. Consistent with this, the oxygen consumption rate (OCR), which reflects mitochondrial metabolic activity, was much lower in unstimulated and −Arg T cells (Figure 7H). Glycolytic activity, measured by the extracellular acidification rate (ECAR; Figure 7H), was also significantly reduced. Overall, −Arg T cells behaved more similarly to quiescent, unstimulated T cells, with reduced respiratory capacity (Figure S7H). Analysis of transcriptomic differences under arginine starvation (Figure 1D) demonstrated downregulation of genes associated with glycolysis/gluconeogenesis, TCA cycle, and the pentose phosphate pathway (Figure S7I; Table S2). Thus, metabolic perturbation may be driven in part by an inability to upregulate metabolic gene expression, suggesting a link to the disruption of chromatin remodeling.

## Discussion

Nutritional stress is used by cancer cells to generate an immunosuppressive microenvironment to impact the function of tumor-infiltrating lymphocytes (Friberg et al., 2002; Mellor and Munn, 2003; Timosenko et al., 2016; Uyttenhove et al., 2003), for example by enzyme-mediated degradation of arginine. Analysis of murine pancreatic adenocarcinoma (PDAC) tissue, which is associated with *ARG2* and *ASS1* upregulation (Zaytouni et al., 2017), found a dramatic reduction in arginine levels in the interstitial fluid (Sullivan et al., 2019). Similarly, reduced levels of plasma arginine (Mussai et al., 2015, 2019) and elevated ARG2 (Mussai et al., 2013) are found in AML patients, suggesting that some tumors can tolerate arginine starvation *in vivo*.

A tumor microenvironment restrictive to T cells must be accompanied by an adaptive mechanism to permit tumor growth. Arginine starvation strongly upregulated the arginine biosynthesis gene *ASS1* in cell lines derived from multiple cancers. These cells proliferated in the absence of arginine when supplemented with the ASS1 substrate citrulline, demonstrating the capacity to import and generate arginine from this precursor. We found *ASS1* upregulation in primary AML blasts, and *ASS1* overexpression has been reported in a number of other cancers (Bateman et al., 2017; Henriet et al., 2017; Rho et al., 2008; Shan et al., 2015a, 2015b; Szlosarek et al., 2007; Tsai et al., 2018), suggesting clinical relevance. Extracellular citrulline in the plasma of AML patients is at concentrations sufficient for THP1 cell proliferation. Citrulline generation/release into the bloodstream has also been observed in mice treated with pegylated arginase I (Fletcher et al., 2015), suggesting that it may be a consequence of increased arginine hydrolysis. We note that the availability of citrulline in patient plasma does not necessarily mean it is taken up by cells; elevated serum levels may be a consequence of reduced uptake. A definitive answer on this issue will require comprehensive analysis of paired serum and intracellular citrulline and arginine levels, alongside *ARG2* and *ASS1* expression, in both tumor and T cells in an *in vivo* setting. However, our work, as well as that of others (Werner et al., 2017), demonstrates that tumor and T cells can import citrulline and use it as an arginine substitute *in vitro* (when *ASS1* is expressed).

*ASS1* upregulation is both necessary and sufficient for citrulline-dependent proliferation in the absence of arginine. Induction was barely detectable in T cells, but T cells genetically engineered to express *ASS1* acquired the ability to proliferate in arginine-free medium using citrulline. This is particularly significant in the context of adoptive T cell therapy, especially given the role of arginine in enhancing the survival and anti-tumor capacity of stimulated T cells (Geiger et al., 2016). CAR-T cell function is disrupted by arginine depletion (Mussai et al., 2019), and engineered expression of *ASS1* enhances their proliferation and *in vivo* cancer targeting behavior (Fultang et al., 2020). It is noteworthy that, unlike human T cells, mouse T cells express *ASS1* and can use citrulline for growth (Fletcher et al., 2015; Lange et al., 2017; Tarasenko et al., 2015), meaning that care is required in interpreting arginine-deprivation murine experiments in the context of human disease.

Upregulation of *ASS1* is dependent on ATF4, which drives transcriptional changes in response to amino acid starvation (Harding et al., 2003; Ye et al., 2010). We identified an enhancer within *ASS1* that is bound by ATF4 and CEBPβ. Deletion of this binding site, or *ATF4* KD, abrogated *ASS1* expression and the ability to use citrulline to proliferate in arginine-free conditions. Although ATF4 protein levels are comparable in activated T cells and THP1 cells, the ability of ATF4 to activate *ASS1* differs in each cell type. ATF4 and CEBPβ failed to bind at the *ASS1* enhancer in arginine-starved T cells, and binding under low arginine conditions did not induce strong transcription, restricted by poor chromatin accessibility and repressive histone methylation.

Arginine starvation of T cells resulted in genome-wide restriction of ATF4/CEBPβ binding, with a reduction in both frequency and strength of binding. The decreases correlated with reduced chromatin accessibility and increased H3K27me3 genome-wide, suggesting this effect may not be limited to just these TFs. This appears to be a direct consequence of increased histone methylation, as treatment of cells with the demethylase inhibitor 2HG produced the same disruption of ATF4/CEBPβ binding at *ASS1* as arginine starvation. This could be a common T cell response to environmental changes, where multiple genes are regulated by introduction of a repressive chromatin environment to restrict TF accessibility (Bevington et al., 2016; Gray et al., 2017; Philip et al., 2017; Sen et al., 2016).

The reduced chromatin accessibility in arginine-starved T cells appears to be associated with the inability of these cells to fully activate. T cells undergo large-scale reprogramming upon stimulation, including metabolic upregulation (Chang et al., 2013; Frauwirth et al., 2002; Gubser et al., 2013; Wang et al., 2011), chromatin reorganization (Bevington et al., 2016), and transcriptional reprogramming (Conley et al., 2016), as well as changes in histone methylation (Araki et al., 2009; Durek et al., 2016; Manna et al., 2015; Russ et al., 2014), which are required for successful activation, effector function, and proliferation. The ATAC-seq signature of arginine-starved T cells was more similar to unstimulated T cells, in line with evidence linking dysfunction in T cell activation and exhaustion to chromatin reorganization (Bevington et al., 2016; Gray et al., 2017; Philip et al., 2017; Sen et al., 2016). Similarly, arginine-starved T cells could not drive the global changes in metabolism associated with activation. This makes a clear counterpoint to the increased metabolism produced following arginine supplementation (Geiger et al., 2016), suggesting that arginine availability plays a key role in regulating metabolic activity. Thus, in the absence of arginine, T cells initiate the process but fail to fully activate. This may explain the immunosuppressive consequences of tumor-mediated arginine degradation.

Although our work does not provide an explanation for the large-scale chromatin defects observed upon arginine starvation, it is likely that no single pathway mediates this effect. However, the observed increase in repressive histone methylation may provide an insight into why these T cells fail to activate properly. Global H3K27me3 levels have been linked to various aspects of T cell function, including differentiation (Araki et al., 2009; Durek et al., 2016; Manna et al., 2015; Russ et al., 2014) and inflammation (Cribbs et al., 2020), and elevated H3K27me3 levels are associated with impaired immunity and more severe disease in COVID-19 (Thompson et al., 2020). In the present study, we provide a link between amino acid deprivation and changes in histone methylation, but further work is required to elucidate what drives this effect.

Arginine has gained significant traction in the context of tumor metabolism and immunotherapy. Our findings suggest multiple therapeutic approaches for *ASS1*-expressing cancers. Arginine supplementation (Geiger et al., 2016) and/or inhibition of arginase (Miret et al., 2019; Steggerda et al., 2017; Timosenko et al., 2017) may relieve the immunosuppression of arginine starvation and reduce T cell dysfunction (Mussai et al., 2013, 2019). Targeted inhibition of ASS1, or adoptive T cell therapy to induce *ASS1* expression in patient immune cells (Fultang et al., 2020), could reduce the tumor proliferative advantage. Arginine depletion is being trialed as a therapeutic strategy for many arginine auxotrophic cancers (Fultang et al., 2016). Importantly, our work and that of others (Fletcher et al., 2015) indicate that this depletion needs to be balanced with the detrimental effect on T cell function. Emphasis should be placed on personalizing treatment according to the *ASS1* status of the tumor.

## STAR★Methods

### Key resources table

**Table:** 

REAGENT or RESOURCE	SOURCE	IDENTIFIER
**Antibodies**		

Rabbit monoclonal anti-ASS1	Abcam	Cat#ab170952; clone EPR12398
Rabbit polyclonal anti-ATF4	Cell Signaling Technology	Cat#11815; RRID:AB_2616025
Rabbit monoclonal anti-Phospho-S6 Ribosomal Protein (Ser235/236)	Cell Signaling Technology	Cat#2211; RRID:AB_331679
Mouse monoclonal anti-GAPDH	Santa Cruz Biotechnology	Cat#sc-32233; RRID:AB_627679; clone 6C5
Rabbit polyclonal anti-CEBPβ	Bethyl	Cat#A302-738A; RRID:AB_10627809
Rabbit polyclonal anti-H3K9me3	Abcam	Cat#ab8898; RRID:AB_306848
Rabbit polyclonal anti-H3K27me3	Millipore	Cat#07-449; RRID:AB_310624
Rabbit polyclonal anti-H3K4me3	Active Motif	Cat#39159; RRID:AB_2615077
Rabbit polyclonal anti-H3K27ac	Diagenode	Cat#C15410196; RRID:AB_2637079
Mouse monoclonal PE/Dazzle™ 594 anti-human CD3 antibody	BioLegend	Cat#317345; RRID:AB_2565850; clone OKT3
Mouse monoclonal FITC anti-CD4 antibody	BD Biosciences	Cat#560132; RRID:AB_2737607; clone RPA-T4
Mouse monoclonal PE anti-CD25 antibody	BD Biosciences	Cat#560132; RRID:AB_2737607; clone M-A251
Mouse monoclonal Brilliant Violet 421™ anti-human CD127 (IL-7Ralpha) antibody	BioLegend	Cat#351309; RRID:AB_10898326; clone A019D5
Mouse monoclonal APC/Cyanine7 anti-human CD197 (CCR7) antibody	BioLegend	Cat#353211; RRID:AB_10915272; clone G043H7
Mouse monoclonal Brilliant Violet 711™ anti-human CD45RA antibody	BioLegend	Cat#304138; RRID:AB_2563815; clone HI100
Mouse monoclonal PE anti-human IFN-gamma antibody	BioLegend	Cat#506507; RRID:AB_315440; clone B27
Rat monoclonal anti-IFN gamma	Thermo Fisher Scientific	Cat#16-7312-81; RRID:AB_469244; clone R4-6A2
Rat monoclonal anti-IFN gamma, biotinylated	Thermo Fisher Scientific	Cat#13-7311-81; RRID:AB_466936; clone XMG1.2

**Biological samples**		

Leukocyte cones	NHS Blood and Transplant, Bristol, UK	N/A
Peripheral blood and bone marrow from healthy donors and AML patients	This paper	N/A

**Chemicals, peptides, and recombinant proteins**		

RPMI-1640 medium for SILAC	Thermo Fisher Scientific	Cat#88365
DMEM for SILAC	Thermo Fisher Scientific	Cat#88364
L-Lysine	Sigma-Aldrich	Cat#L5501; CAS: 56-87-1
L-Arginine	Sigma-Aldrich	Cat#A5006; CAS: 74-79-3
L-Citrulline	Sigma-Aldrich	Cat#C7629; CAS: 372-75-8
Rapamycin	Sigma-Aldrich	Cat#553210; CAS: 53123-88-9
BCH (2-amino-2-norbornanecarboxylic acid)	Sigma-Aldrich	Cat#A7902; CAS: 20448-79-7
GPNA (L-γ-Glutamyl-p-nitroanilide)	Sigma-Aldrich	Cat#G1135; CAS: 7300-59-6
(2S)-Octyl-alpha-hydroxyglutarate	Cayman Chemical	Cat#16367; CAS: 1391194-64-1
Brefeldin A Solution (1,000X)	BioLegend	Cat#420601
Paraformaldehyde	Sigma Aldrich	Cat#P6148; CAS: 30525-89-4
Di(N-succinimidyl) glutarate	Sigma Aldrich	Cat#50424-50MG-F; CAS: 79642-50-5
Critical Commercial Assays		
CD4 MicroBeads, human	Miltenyi Biotec	Cat#130-045-101
CD8^+^ T Cell Isolation Kit, human	Miltenyi Biotec	Cat#130-096-495
Dynabeads Human T-Activator CD3/CD28 for T Cell Expansion and Activation	Thermo Fisher Scientific	Cat#11131D
LIVE/DEAD Fixable Aqua Dead Cell Stain Kit, for 405 nm excitation	Thermo Fisher Scientific	Cat#L34966
CellTrace CFSE Cell Proliferation Kit, for flow cytometry	Thermo Fisher Scientific	Cat#C34570
CellTrace Violet Cell Proliferation Kit, for flow cytometry	Thermo Fisher Scientific	Cat#C34571
eBioscience Foxp3 / Transcription Factor Fixation/Permeabilization Concentrate and Diluent	Thermo Fisher Scientific	Cat#00-5521-00
AccQ-Tag Ultra Derivatization Kit	Waters	Cat#186003836
Seahorse XF RPMI medium, pH 7.4, 500 mL	Agilent Technologies	Cat#103576-100
Seahorse XF Cell Energy Phenotype Test Kit	Agilent Technologies	Cat#103275-100
RNeasy Mini kit	QIAGEN	Cat#74104
HumanHT-12 v4.0 Expression BeadChip Kit	Illumina	Cat#BD-103-0604
SuperScript III Reverse Transcriptase	Thermo Fisher Scientific	Cat#18080044
Lipofectamine RNAiMAX Transfection Reagent	Thermo Fisher Scientific	Cat#13778150
MethoCult H4100	StemCell Technologies, Inc.	Cat#04100
TA Cloning Kit, with pCR2.1 Vector, without competent cells	Thermo Fisher Scientific	Cat#K202040
L-Citrulline photometric assay	Immundiagnostik	Cat#K6600
DNeasy Blood & Tissue Kit	QIAGEN	Cat#69504
EZ DNA Methylation-Lightning Kit	Zymo Research	Cat#D5030
ZymoTaq DNA Polymerase	Zymo Research	Cat#E2001
Dynabeads Protein A for Immunoprecipitation	Thermo Fisher Scientific	Cat#10002D
Dynabeads Protein G for Immunoprecipitation	Thermo Fisher Scientific	Cat#10004D
Pierce ChIP-Grade Protein A/G Plus Agarose	Thermo Fisher Scientific	Cat#26159
QIAquick PCR purification kit	QIAGEN	Cat#28106
NEBNext Ultra II DNA library preparation kit for Illumina	NEB	Cat#E7645S
Illumina Tagment DNA Enzyme and Buffer Small Kit	Illumina	Cat#20034197
MinElute PCR Purification Kit	QIAGEN	Cat#28004
KAPA Library Quantification Kit	Roche	Cat#07960140001
NextSeq 500 High Output Kit (75 cycles)	Illumina	Cat#FC-404-1005

**Deposited data**		

Raw and analyzed data	This paper	GEO: GSE137034
ChIP-seq data for histone modifications in THP1 cells	Godfrey et al., 2019	GEO: GSE117865
ChIP-seq data for histone modifications in HeLa cells	Kuznetsova et al., 2015	GEO: GSE61911
AML patient RNA-seq data	Quek et al., 2016	ArrayExpress: E-MTAB-2672

**Experimental models: Cell lines**		

Human: THP1 cells	ATCC	Cat#TIB-202; RRID:CVCL_0006
Human: NB4 cells	Cancer Research UK Centre	RRID:CVCL_0005
Human: HL60 cells	ATCC	Cat#CCL-240; RRID:CVCL_0002
Human: MOLM13 cells	DSMZ	Cat# ACC-554; RRID:CVCL_2119
Human: OCI-AML3 cells	DSMZ	Cat#ACC-582; RRID:CVCL_1844
Human: RT112 cells	Cancer Research UK Centre	RRID:CVCL_1670
Human: LNCaP cells	Cancer Research UK Centre	RRID:CVCL_0395
Human: HeLa cells	Cancer Research UK Centre	RRID:CVCL_0030
Human: RS4;11 cells	ATCC	Cat#CRL-1873; RRID:CVCL_0093

**Oligonucleotides**		

ChIP-qPCR primers	See Table S4 for sequences	N/A
Taqman qRT-PCR probes	Thermo Fisher Scientific; See Table S4 for probe IDs	Cat# 4331182
ON-TARGETplus SMARTpool siRNA targeting *ASS1*	Dharmacon	Cat#L-010257-00-0005
ON-TARGETplus SMARTpool siRNA targeting *ATF4*	Dharmacon	Cat#L-005125-00-0005
ON-TARGETplus SMARTpool siRNA non-targeting control pool	Dharmacon	Cat#D-001810-10-20
*ASS1* enhancer gRNA1 forward sequence: CACCCTGCCCTCCCAGCAGCGGGT	This paper	N/A
*ASS1* enhancer gRNA1 reverse sequence: AAACACCCGCTGCTGGGAGGGCAG	This paper	N/A
*ASS1* enhancer gRNA2 forward sequence: CACCATTTCTCTGCATGCATACAC	This paper	N/A
*ASS1* enhancer gRNA2 reverse sequence: AAACGTGTATGCATGCAGAGAAAT	This paper	N/A
Bisulfite sequencing amplification primers	See Table S4 for sequences	N/A

**Recombinant DNA**		

pSpCas9(BB)-2A-GFP	Addgene	Cat#PX458; RRID:Addgene_48138
ASS1 Lentiviral Vector (Human) (CMV) (pLenti-GIII-CMV-GFP-2A-Puro)	abm	Cat#LV082170-ABM

**Software and algorithms**		

Progenesis QI	Waters	https://www.waters.com/waters/en_US/Progenesis-QI-Software/nav.htm?cid=134790655&locale=en_US
Seahorse XF Cell Energy Phenotype Test Report Generator	Agilent Technologies	https://www.agilent.com/en/product/cell-analysis/real-time-cell-metabolic-analysis/xf-software/seahorse-xf-cell-energy-phenotype-test-report-generators-740898
GenomeStudio	Illumina	https://www.illumina.com/techniques/microarrays/array-data-analysis-experimental-design/genomestudio.html
MassHunter Workstation, vB8.0	Agilent Technologies	https://www.agilent.com/en/product/software-informatics/mass-spectrometry-software
ImageJ v2.1.0/1.53c	Schindelin et al., 2012	https://imagej.nih.gov/ij/index.html
NGseqBasic VS20.0	Telenius and Hughes, 2018	https://github.com/Hughes-Genome-Group/NGseqBasic/releases
fastQC v0.11.9	N/A	https://www.bioinformatics.babraham.ac.uk/projects/fastqc/
bowtie v1.2.3	Langmead et al., 2009	https://sourceforge.net/projects/bowtie-bio/
trim_galore v0.6.5	N/A	https://www.bioinformatics.babraham.ac.uk/projects/trim_galore/
samtools v1.10	Li et al., 2009	http://www.htslib.org
bedtools v2.29.2	Quinlan and Hall, 2010	https://bedtools.readthedocs.io/en/latest/
Homer v4.8	Heinz et al., 2010	http://homer.ucsd.edu/homer/
MACS2 v2.2.7.1	Zhang et al., 2008	https://github.com/macs3-project/MACS
Diffbind v3.0.4	Ross-Innes et al., 2012	http://bioconductor.org/packages/release/bioc/html/DiffBind.html
MEME Suite	Grant et al., 2011	https://meme-suite.org/meme/
EdgeR v3.26.5	Robinson et al., 2010	https://bioconductor.org/packages/release/bioc/html/edgeR.html
PRISM 9	GraphPad	https://www.graphpad.com
UCSC Genome Browser	Kent et al., 2002	https://genome.ucsc.edu/index.html

### Resource availability

#### Lead contact

Further information and requests for resources and reagents should be directed to and will be fulfilled by the lead contact, Thomas Milne (thomas.milne@imm.ox.ac.uk).

#### Materials availability

All unique/stable reagents generated in this study are available from the Lead Contact with a completed Materials Transfer Agreement.

#### Data and code availability

All high throughput data have been deposited in the Gene Expression Omnibus (GEO) under accession number GSE137034.

### Experimental model and subject details

#### Human samples

Human leukocyte cones were purchased from NHS Blood and Transplant (Bristol, UK). Peripheral blood and bone marrow samples were obtained from healthy donors (n = 10; 50% male/female; age range 34-72, median 57.5) and AML patients (n = 16; age range 30-77, median 64.5) and processed on the same day. All volunteers gave written informed consent in accordance to the declaration of Helsinki. Data and sample collection were approved by the Oxford Research Ethics Committee (MDSBio Study, MREC 06/Q1606/110; Oxford Musculoskeletal Biobank, MREC 09/H0606/11: South Central Oxford C Research Ethics Committee and as part of the AML17 clinical trial (MREC 08/MRE09/29: Wales Research Ethics Committee)).

#### Cell lines

Human tumor cell lines NB4 (female; Cancer Research UK; RRID CVCL_0005), MOLM13 (male; DSMZ, ACC-554; RRID CVCL_2119), RT112 (female; CRUK; RRID CVCL_1670), LNCaP (male; CRUK; RRID CVCL_0395), OCI-AML3 (male; DSMZ, ACC-582; RRID CVCL_1844), THP1 (male; ATCC, TIB-202; RRID CVCL_0006), HL60 (female; ATCC, CCL-240; RRID CVCL_0002) and RS4;11 (female; ATCC, CRL-1873; RRID CVCL_0093) cells were cultured in RPMI-1640 supplemented with 10% fetal calf serum and GlutaMAX (ThermoFisher Scientific). HeLa cells (female; CRUK; RRID CVCL_0030) were cultured in DMEM supplemented with 10% fetal calf serum and GlutaMAX (ThermoFisher Scientific). All cells were grown at 37°C in a humidified incubator with 5% CO_2_.

### Method details

#### Cell culture

For arginine starvation experiments, RPMI-1640 or DMEM medium for SILAC (ThermoFisher Scientific) was used, supplemented with 10% FCS, GlutaMAX and 40 mg/L L-lysine. For low arginine conditions, the medium was further supplemented with 20 μM L-arginine. Standard RPMI-1640 medium, supplemented with 10% FCS and GlutaMAX, was used as complete (+Arg) medium. Where indicated, citrulline was added at a concentration of 0.4 mM. Rapamycin (Merck) was used at 20 nM. BCH (2-amino-2-norbornanecarboxylic acid, Sigma Aldrich) and GPNA (L-γ-Glutamyl-p-nitroanilide, Sigma Aldrich) were used at 2 mM and 1 mM, respectively. For 2-hydroxyglutarate work, T cells were treated with (2S)-Octyl-alpha-hydroxyglutarate (Cayman Chemical) at 500 μM immediately after stimulation, then supplemented with fresh compound after 48h, prior to harvest at 72h.

#### Isolation of human T cells

Peripheral blood mononuclear cells (PBMCs) were isolated from commercially available leukocyte cones (NHS Blood and Transplant, Bristol) by density gradient centrifugation. CD4+ and CD8+ T cells were purified from PBMCs by positive selection using magnetic cell separation with human CD4 MicroBeads (130-045-101, Miltenyi Biotec) or human CD8+ T Cell Isolation Kit (Miltenyi Biotec), respectively. Cells were stimulated by addition of 4 μL/10^6^ cells anti-human CD3/CD28 antibody-conjugated beads (Dynabeads Human T-Activator CD3/CD28, 11131D, Thermofisher Scientific).

#### Flow cytometry

Cell viability was determined using LIVE-DEAD Fixable Aqua Dead Cell Stain (ThermoFisher Scientific). Cellular proliferation was monitored by labeling cells with either CellTrace CFSE (ThermoFisher Scientific) or CellTrace Violet (CTV; ThermoFisher Scientific) dyes. For IFNγ FACS analysis, cells were treated with Brefeldin A (Biolegend) for 4h prior to fixation and permeabilization using the eBioscience Foxp3/Transcription Factor Fixation/Permeabilization buffer set (ThermoFisher Scientific). FACS analysis was performed using an LSR Fortessa flow cytometer (BD) or a Cyan ADP flow cytometer (Dakocytomation). FACS sorting was performed using the Aria III SORP machine (BD).

#### ELISA measurement of IFNγ levels

IFNγ levels in human CD4+ T cell culture supernatants were measured using a sandwich ELISA assay. Briefly, the anti-IFNγ capture antibody was diluted in coating buffer (100 mM NaHCO_3_ pH 7.9) to give a concentration of 2 μg/mL. 25 μL was used to coat each well of a 96-well enhanced binding ELISA plate (Greiner). The plate was covered and incubated at 4°C overnight. The following day, the plate wells were washed 6 times with washing buffer (PBS with 0.5% Tween-20) and blocked for 2h using blocking buffer (PBS with 10% FCS) at 200 μL per well. IFNγ standards (Peprotech) were used to generate a standard curve. 50 μL samples were added and incubated at 4°C overnight. Plates were washed 6 times and 25 μL 1 μg/mL biotinylated anti-IFNγ detection antibody added to each well for 1h, then washed 8 times. 25 μL 2.5 μg/mL avidin-peroxidase (Sigma Aldrich) was added to each well and incubated at room temperature for 40 min. The plate was washed 8 times with washing buffer before the ABTS substrate and H_2_O_2_ mixture was added. The reaction was allowed to develop for 10-80 min based on the intensity of color changes before stopping with 2 M H_2_SO_4_. Absorbance was read at 405 nM using a spectrophotometer (BMG SPECTROstar plate reader). A 4-parameter standard curve was made using the MARS data analysis software (BMG) and used to infer the concentration of cytokine in each well.

#### Plasma citrulline measurements

Samples were obtained from healthy donors and AML patients and processed on the same day. All volunteers gave written informed consent in accordance to the declaration of Helsinki. Data and sample collection were approved by the Oxford Research Ethics Committee (MDSBio Study, MREC 06/Q1606/110; Oxford Musculoskeletal Biobank, MREC 09/H0606/11: South Central Oxford C Research Ethics Committee and as part of the AML17 clinical trial (MREC 08/MRE09/29: Wales Research Ethics Committee)). Plasma from peripheral blood or bone marrow was isolated following application of the Ficoll-Hypaque density centrifugation method in the process of deriving multiple elemental components of blood. Briefly, samples were diluted in R10 medium at a 1:1 volume ratio. 30 mL of this was overlaid carefully onto 15 mL of Lymphoprep (AxisShield) and centrifuged for 30 min at 300 g without brake or acceleration. Following centrifugation, plasma from AML patients or healthy controls was collected by aspirating the upper layer of the suspension. Human plasma citrulline was quantified by photometric assay (K6600; Immundiagnostik).

#### Intracellular amino acid measurements

THP1 cells were incubated in the indicated media for 72h, then pelleted and stored at −80°C. For analysis, the cell pellet was warmed to 0°C, 400 μL of 80% (vol/vol) methanol in water (−80°C) was added, and the samples vortexed gently for 30 min at room temperature. The mixture was incubated for 60 min at −80°C, centrifuged at 14,000 g for 10 min at 4°C to pellet the cell debris and the metabolite-containing supernatant transferred to a new tube. 400 μL 80% (vol/vol) methanol in water (−80°C) was added to the remaining pellet. The resulting mixture was vortexed for 5 min at room temperature, centrifuged at 14,000 g for 10 min at 4°C and the supernatant transferred to the tubes containing previous supernatant. Combined supernatants were dried in a SpeedVac. The dried samples were stored at −80°C until analysis. The extracts were resuspended in 20 μL water (LC-MS grade, Merck) with 0.1% formic acid (FA, LC-MS grade, Fisher Scientific) per 5 million cells by shaking at 1200 rpm for 30 min, then centrifuged at 2000 g for 2 min.

The supernatant was analyzed by liquid chromatography-mass spectrometry (LC-MS), consisting of an Agilent 1290 Infinity Ultra-High Performance Liquid Chromatography system (UHPLC) equipped with a quaternary pump delivery system (G4204A), a HiP autosampler (G4226A), a column thermostat (G1316C). An ACQUITY Glycoprotein Amide Column, (300Å, 1.7 μm, 2.1 mm × 150 mm; Waters) was used. The UHPLC system was coupled to a 6560 Ion mobility QTOF LC/MS mass spectrometer (Agilent Technologies) equipped with a Jetstream ESI-AJS source.

The data were acquired by LC-MS in QToF mode using positive electrospray ionization (ESI+). Two reference ions, m/z 121.0508 and 922.0097, were used as internal standards. The Dual AJS ESI settings were as follows: gas temperature: 300°C, the drying gas: 8 L/min, nebulizer 35 MPa, sheath gas temperature 350°C, sheath gas flow 11 L/ min, Vcap 3.500 V and nozzle voltage 1000 V. The fragmentor of the mass spectrometer TOF was set to 400 V.

The UHPLC gradient was composed of 20% buffer A (water (0.1% FA)) and 80% buffer B (Acetonitrile (LC-MS graded from Merck) (0.1% FA)) with a flow rate of 0.30 mL; 0-8 min 20% −35% A; 8-9 min 35% −20% A. The gradient was followed by a 3 min post-time to re-equilibrate the column. The external standards, L-arginine monohydrochloride and L-citrulline (Sigma-Aldrich), were used for identification. The raw LC-MS data was processed and analyzed using the MassHunter Workstation software package (Agilent Technologies, version B8.0).

#### Gene expression analysis

For microarray analysis, RNA was extracted from THP1 or stimulated human CD4+ T cells using the RNeasy Mini kit (QIAGEN). RNA was converted into biotin-labeled cRNA for hybridization. The hybridized and washed chips were scanned using iScan Scanner (Illumina).

For quantitative reverse transcription PCR, RNA was extracted using the RNeasy Mini kit (QIAGEN) and contaminating DNA digested on the column. cDNA was generated with SuperScript III reverse transcriptase (ThermoFisher Scientific) using random hexamer primers. Taqman probes (ThermoFisher Scientific) used for qPCR analysis are detailed in Table S4.

#### Gene knockdown

Knockdown of *ASS1* and *ATF4* were mediated using ON-TARGETplus SMARTpool siRNA (Dharmacon). The siRNA used were: *ASS1* (L-010257-00-0005); *ATF4* (L-005125-00-0005). ON-TARGETplus Non-targeting Control Pool (D-001810-10-20) was used as the control. THP1 cells were electroporated as described previously (Kerry et al., 2017). HeLa cells were transfected using Lipofectamine RNAi-MAX (ThermoFisher Scientific) following the manufacturer’s protocol. After transfection, cells were left to recover for 6h, then counted and transferred to the indicated incubation media.

#### Transgenic expression of ASS1

Purified human CD8+ T cells were labeled with 5 μM cell tracker violet (CTV; ThermoFisher Scientific) and transduced with lentiviral vector coexpressing human *ASS1* and *GFP* (pLenti-GIII-CMV-GFP-2A-Puro, abm). After 24 h cells were stimulated with anti-CD3/anti-CD28 beads (ThermoFisher Scientific), then left for a further 24 h. Cells were PBS-washed then transferred to selection medium (standard growth medium, arginine-free medium and arginine-free medium supplemented with 0.4 mM citrulline). Medium was replenished 48 h later to ensure T cells were not starved of any nutrients except arginine in the appropriate cultures. After 96 h in selection medium, cells were analyzed by flow cytometry for CTV and GFP expression, gating on single, live CTV+ CD8+ T cells.

#### CRISPR-Cas9-mediated mutation

Deletion of the ATF4-binding motif within *ASS1* in THP1 cells was achieved with the pSpCas9(BB)-2A-GFP plasmid (PX458; Addgene); THP1 cells were cotransfected with two plasmids containing guide RNAs flanking the ATF4 binding motif (sequences shown in Figure 3C). GFP-positive cells were isolated by FACS after 24 h and plated on Methocult H4100 (Stem Cell Technologies) to isolate clones. Clones were screened by PCR of the targeted region, individual alleles were cloned into the TA-cloning vector pCR2.1 (ThermoFisher Scientific) and deletions confirmed by Sanger sequencing.

#### Western blotting analysis

Proteins were extracted from cells using a high-salt lysis buffer (20 mM Tris-HCl pH 8.0, 300 mM KCl, 5 mM EDTA, 20% glycerol, 0.5% IGEPAL CA-630, protease inhibitor cocktail), and western blotting analysis was performed as previously described (Wilkinson et al., 2013). Membranes were visualized using the Odyssey Imaging System (Li-Cor) or by enhanced chemiluminescence (ECL). Quantification was conducted using ImageJ (Schindelin et al., 2012).

#### Bisulfite sequencing

DNA was purified from cells using the DNeasy Blood & Tissue kit (QIAGEN), then bisulfite treated using the EZ DNA Methylation-Lightning kit (Zymo Research). DNA was amplified using ZymoTaq polymerase (Zymo Research), then cloned into the TA-cloning vector pCR2.1 (ThermoFisher Scientific). At least 12 individual colonies were sequenced per sample and compared to the non-bisulfite-treated sequence to identify methylation status.

#### Chromatin Immunoprecipitation

ChIP and ChIP-seq experiments were carried out as previously described (Kerry et al., 2017; Wilkinson et al., 2013). Briefly, cells were single-fixed (1% formaldehyde for 10 min) for histone ChIP or double-fixed (2 mM disuccinimidyl glutarate for 30 min, then 1% formaldehyde for 30 min) for TF ChIP. Fixed samples were sonicated using a Covaris (Woburn, MA) to generate 200-300 bp fragments. Antibody-chromatin complexes were isolated using a mixture of protein A and G agarose beads (histone ChIP; ThermoFisher Scientific) or protein A and G dynabeads (TF ChIP; ThermoFisher Scientific). Bound beads were washed three times with RIPA buffer (50 mM HEPES-KOH, pH 7.6, 500 mM LiCl, 1 mM EDTA, 1% NP-40, and 0.7% Na deoxycholate) and once with Tris-EDTA. Crosslinks were reversed at 65°C overnight, then samples were RNase A- and proteinase K-treated and DNA was purified using a QIAquick PCR purification kit (QIAGEN). For ChIP-qPCR samples were quantified relative to input DNA. For ChIP-seq, DNA libraries were generated using the NEBNext Ultra II DNA library preparation kit for Illumina (NEB) and quantified using the KAPA Library Quantification Kit (Roche). Samples were sequenced by paired-end sequencing on a NextSeq 500 (Illumina). For reference-normalized ChIP-seq (Orlando et al., 2014), fixed *Drosophila melanogaster* S2 cells were added to fixed cells, and the ChIP protocol was followed as normal. Sequencing reads from input and IP samples were mapped to hg19 and dm3 genome builds, and the ratio of dm3:hg19 reads in input and IP samples was used to adjust hg19 read counts.

#### ATAC-seq

The ATAC-seq protocol was adapted from Buenrostro et al. (2015). After incubation for 72h in the indicated media, 5x10^4^ THP1 or CD4+ human T cells were washed with PBS, then resuspended in cold lysis buffer (10 mM Tris-HCl, pH 7.4, 10 mM NaCl, 3 mM MgCl_2,_ 0.1% IGEPAL CA-630). Nuclei were pelleted at 500*g* for 10 min, then resuspended in transposase reaction mix (Illumina) and incubated at 37°C for 30 min. DNA was purified using a MinElute kit (QIAGEN). The DNA fragments were amplified in a 12-cycle PCR to add unique indices for sequencing and purified using a MinElute kit. Samples were sequenced by paired-end sequencing on a NextSeq 500 (Illumina).

#### Metabolomics

##### Sample preparation

Cell pellets were snap frozen with liquid N_2_ to arrest metabolic processes and cells lysed with the addition of ice-cold 80% methanol_(aq)_ at a concentration of 1x10^6^ cells/100 μL to extract metabolites into solution. Metabolite extracts were centrifuged at 21,693 g for 20 min to remove cell debris and filtered using 10 kDa MWCO filters (Merck Millipore) prior to analysis. Metabolomics analysis was performed on three separate hyphenated platforms comprising anion-exchange chromatography-tandem mass spectrometry and reversed phase-chromatography mass spectrometry of derivatized and underivatized cell extracts.

##### Ion chromatography-tandem mass spectrometry

Anion exchange chromatography-tandem mass spectrometry was performed using a Dionex ICS-5000+ Capillary HPIC system (Thermo Scientific) coupled to a Q Exactive hybrid quadrupole-Orbitrap mass spectrometer (Thermo Scientific) as described (Walsby-Tickle et al., 2020).

##### Reversed-phase chromatography-MS

Metabolite extracts were derivatized using an AccQ-Tag Derivatization Kit (Waters). A 5 μL partial loop injection was used for all analyses and chromatographic separation was performed using a Dionex Ultimate 3000 UHPLC system (Thermo Scientific) coupled to a Q Exactive hybrid quadrupole-Orbitrap mass spectrometer (Thermo Scientific). An AccQ-tag Ultra C18 column (2.1 × 100 mm, 1.7 μm; Waters) at 50°C was used with mobile phase A: water with 10% Eluent A concentrate (Waters) and mobile phase B: acetonitrile with 1.3% formic acid. The linear gradient used was: 0 min, 0.1% B; 0.54 min, 9.1% B; 5.74 min 21.2% B; 7.74 min, 59.6% B; 8.04 min, 90% B; 8.05 min, 90% B; 8.64 min, 0.1% B; 9.5 min, 0.1% B. The flow rate was 0.5 mL/min and the total analyzed time was 9.5 min. The Q Exactive mass spectrometer was equipped with a HESI II probe in positive ion mode with source parameters set as follows: sheath gas flow rate, 50; auxiliary gas flow rate, 20; sweep gas flow rate, 3; spray voltage, 3.7 kV; capillary temperature, 300°C; S-lens RF level, 70 and heater temperature 250°C. Scan parameters were set as follows: in-source CID, 0.0 eV; microscans, 1; resolution, 70,000; AGC target, 3 × 10^6^ ions; maximum IT, 200 ms; scan range, 70-1050 *m/z*.

##### Reversed-phase chromatography tandem MS

A 5 μL partial loop injection was used for all analyses and chromatographic separation was performed using a Dionex Ultimate 3000 UHPLC system (Thermo Scientific) coupled to a Q Exactive hybrid quadrupole-Orbitrap mass spectrometer (Thermo Scientific). A CORTECS T3 C18 column (2.1 × 100 mm, 1.6μm; Waters, Milford, MA, USA) at 40°C was used with mobile phase A: water with 0.1% formic acid and mobile phase B: methanol with 0.1% formic acid. The linear gradient used was: 0 min, 5% B; 4.0 min, 50% B; 12.0 min 99.9% B; 14.0 min, 99% B; 15.1 min, 5% B. The flow rate was 0.3 mL/min and the total analyzed time was 18 min. The Q Exactive mass spectrometer was equipped with a HESI II probe in negative ion mode with source parameters set as follows: sheath gas flow rate, 25; auxiliary gas flow rate, 8; sweep gas flow rate, 0; spray voltage, 3.5 kV; capillary temperature, 300°C; S-lens RF level, 70 and heater temperature 300°C. Scan parameters were set as follows: in-source CID, 0.0 eV; microscans, 1; resolution, 70,000; AGC target, 5 × 10^6^ ions; maximum IT, 120 ms; scan range, 60-900 *m/z*. dd-MS^2^ parameters were set at follows: microscans, 2; resolution, 17500; AGC target, 5 × 10^4^ ions; minimum AGC target, 2.5 × 10^3^ ions; maximum IT, 80 ms; loop count, 10; MSX count, 1; topN, 10; isolation window, 2.0 *m/z*; collision energy, 35; charge exclusion, 3-8, > 8 and dynamic exclusion, 30.0 s.

#### Seahorse metabolic analysis

CD4+ T cells were cultured as indicated for 72 h, after which they were washed and resuspended in Seahorse XF RPMI medium (Agilent Technologies; pH 7.4, supplemented with 1 mM HEPES, 25 mM glucose, 1 mM pyruvate, 2 mM glutamine) at 5x10^6^ cells/mL. 2x10^5^ cells were added to each well of a 96-well assay plate, pre-coated with poly-D-lysine. Each sample was added to four wells to provide technical replicates. Mitochondrial respiration was measured by oxygen consumption rate (OCR) and glycolytic rate by extracellular acidification rate (ECAR), using a Seahorse XFe96 Extracellular Flux Analyzer (Agilent Technologies) with analysis using the Seahorse XF Cell Energy Phenotype report generator. Metabolic activity was measured in the presence of glucose at the indicated time points, under resting conditions (first three data points) and under stress conditions following addition of 1 μM oligomycin and 1 μM carbonyl cyanide 4-(trifluoromethoxy)phenylhydrazone (FCCP; Agilent Technologies).

### Quantification and statistical analysis

#### Metabolomics data processing

Raw mass spectrometry data files were processed using Progenesis QI (Waters, Elstree, UK) to perform retention time (RT) and *m/z* alignment as well as compound-feature identification. Metabolites were identified by comparison of accurate mass, RT, isotopic distribution and fragmentation data provided by authentic standards (Level 1 identifications) and putative metabolite identifications were made using accurate mass, isotopic distribution and *in silico* fragmentation pattern matching where available (Level 2 identifications).

#### Gene expression analysis by microarray

Gene expression analysis was performed using the Human HT12v4.0 Expression Beadchip (Illumina) and GenomeStudio software (Illumina). Samples were quantile normalized with preprocessCore (R) before differential gene expression analysis using limma (R).

#### Next-generation sequencing analysis

Quality control of FASTQ reads, alignment, PCR duplicate filtering, and blacklisted region filtering were performed using the NGseqBasic pipeline (Telenius and Hughes, 2018). Briefly, the quality of the FASTQ files was confirmed with fastQC (https://www.bioinformatics.babraham.ac.uk/projects/fastqc/), then reads were mapped using bowtie (Langmead et al., 2009) against hg19. Unmapped reads were trimmed with trim_galore (https://www.bioinformatics.babraham.ac.uk/projects/trim_galore/) and remapped. Short unmapped reads were combined using Flash and mapped again. PCR duplicates were removed with samtools rmdup (Li et al., 2009), and any reads mapping to Duke blacklisted regions (UCSC) were removed using bedtools (Quinlan and Hall, 2010). Tag directories of sequence reads were generated using the Homer tool makeTagDirectory (Heinz et al., 2010). Bigwigs were generated with the makeBigWig.pl command, normalizing counts to tags per 10 million, and visualized in the UCSC genome browser (Kent et al., 2002). ChIP-seq peaks were called using the Homer tool findPeaks, with the input track provided for background correction, using the -style histone or -style factor option to call peaks of histone modification or TF binding, respectively. ATAC-seq peaks were called using the MACS2 callpeaks command (Zhang et al., 2008), with the -B and -f BAMPE options. Differential ATAC peak analysis was conducted using Diffbind (Ross-Innes et al., 2012) and EdgeR (Robinson et al., 2010); peaks were considered different with FDR < 0.05. Hierarchical clustering and principal component analysis were conducted using the R package stats (commands: hclust, kmeans and prcomp). Dendrograms were generated using a ‘friend of friends’ clustering algorithm. Metagene profiles were generated using the Homer tool annotatePeaks.pl. Motif analysis was conducted using the FIMO function of MEME Suite (Grant et al., 2011), using TF binding profiles from JASPAR (Khan et al., 2018).

#### Statistical analysis

Statistical analysis was conducted using Prism 9 (GraphPad) or R (v3.3.3). Details of tests used, n values and p values are provided in figure legends or STAR methods. All tests were conducted two-tailed. For T cell experiments, n values refer to the number of donors; for cell lines, n values refer to independent replicates. No statistical methods were used to estimate sample sizes.

## Supplemental information

Document S1. Figures S1–S7 and Table S4

Table S1. Gene expression changes associated with arginine starvation in THP1 cells and stimulated human CD4^+^ T cells, related to Figure 1

Table S2. Gene expression changes associated with arginine starvation in stimulated human CD4^+^ T cells associated with selected KEGG pathway terms, related to Figure 1

Table S3. Metabolite levels in human CD4^+^ T cells measured by mass spectrometry, related to Figure 7

Document S2. Article plus supplemental information

## References

[bib1] Ameri K., Harris A.L. (2008). Activating transcription factor 4. Int. J. Biochem. Cell Biol..

[bib2] Araki Y., Wang Z., Zang C., Wood W.H., Schones D., Cui K., Roh T.Y., Lhotsky B., Wersto R.P., Peng W. (2009). Genome-wide analysis of histone methylation reveals chromatin state-based regulation of gene transcription and function of memory CD8^+^ T cells. Immunity.

[bib3] Bar-Peled L., Sabatini D.M. (2014). Regulation of mTORC1 by amino acids. Trends Cell Biol..

[bib4] Bateman L.A., Ku W.M., Heslin M.J., Contreras C.M., Skibola C.F., Nomura D.K. (2017). Argininosuccinate synthase 1 is a metabolic regulator of colorectal cancer pathogenicity. ACS Chem. Biol..

[bib5] Bevington S.L., Cauchy P., Piper J., Bertrand E., Lalli N., Jarvis R.C., Gilding L.N., Ott S., Bonifer C., Cockerill P.N. (2016). Inducible chromatin priming is associated with the establishment of immunological memory in T cells. EMBO J..

[bib6] Bronte V., Zanovello P. (2005). Regulation of immune responses by l-arginine metabolism. Nat. Rev. Immunol..

[bib7] Buenrostro J.D., Wu B., Chang H.Y., Greenleaf W.J. (2015). ATAC-seq: A method for assaying chromatin accessibility genome-wide. Curr. Protoc. Mol. Biol..

[bib8] Carr E.L., Kelman A., Wu G.S., Gopaul R., Senkevitch E., Aghvanyan A., Turay A.M., Frauwirth K.A. (2010). Glutamine uptake and metabolism are coordinately regulated by ERK/MAPK during T lymphocyte activation. J. Immunol..

[bib9] Chang C.H., Curtis J.D., Maggi L.B., Faubert B., Villarino A.V., O’Sullivan D., Huang S.C., van der Windt G.J., Blagih J., Qiu J. (2013). Posttranscriptional control of T cell effector function by aerobic glycolysis. Cell.

[bib10] Chantranupong L., Scaria S.M., Saxton R.A., Gygi M.P., Shen K., Wyant G.A., Wang T., Harper J.W., Gygi S.P., Sabatini D.M. (2016). The CASTOR proteins are arginine sensors for the mTORC1 pathway. Cell.

[bib11] Chowdhury R., Yeoh K.K., Tian Y.M., Hillringhaus L., Bagg E.A., Rose N.R., Leung I.K., Li X.S., Woon E.C., Yang M. (2011). The oncometabolite 2-hydroxyglutarate inhibits histone lysine demethylases. EMBO Rep..

[bib12] Christensen H.N. (1990). Role of amino acid transport and countertransport in nutrition and metabolism. Physiol. Rev..

[bib13] Closs E.I., Simon A., Vékony N., Rotmann A. (2004). Plasma membrane transporters for arginine. J. Nutr..

[bib14] Conley J.M., Gallagher M.P., Berg L.J. (2016). T cells and gene regulation: The switching on and turning up of genes after T cell receptor stimulation in CD8 T cells. Front. Immunol..

[bib15] Cribbs A.P., Terlecki-Zaniewicz S., Philpott M., Baardman J., Ahern D., Lindow M., Obad S., Oerum H., Sampey B., Mander P.K. (2020). Histone H3K27me3 demethylases regulate human Th17 cell development and effector functions by impacting on metabolism. Proc. Natl. Acad. Sci. USA.

[bib16] Delage B., Fennell D.A., Nicholson L., McNeish I., Lemoine N.R., Crook T., Szlosarek P.W. (2010). Arginine deprivation and argininosuccinate synthetase expression in the treatment of cancer. Int. J. Cancer.

[bib17] Dunn G.P., Old L.J., Schreiber R.D. (2004). The immunobiology of cancer immunosurveillance and immunoediting. Immunity.

[bib18] Durek P., Nordström K., Gasparoni G., Salhab A., Kressler C., de Almeida M., Bassler K., Ulas T., Schmidt F., Xiong J., DEEP Consortium (2016). Epigenomic profiling of human CD4^+^ T cells supports a linear differentiation model and highlights molecular regulators of memory development. Immunity.

[bib19] Ensor C.M., Holtsberg F.W., Bomalaski J.S., Clark M.A. (2002). Pegylated arginine deiminase (ADI-SS PEG20,000 mw) inhibits human melanomas and hepatocellular carcinomas in vitro and in vivo. Cancer Res..

[bib20] Esslinger C.S., Cybulski K.A., Rhoderick J.F. (2005). N_γ_-aryl glutamine analogues as probes of the ASCT2 neutral amino acid transporter binding site. Bioorg. Med. Chem..

[bib21] Fiedler T., Strauss M., Hering S., Redanz U., William D., Rosche Y., Classen C.F., Kreikemeyer B., Linnebacher M., Maletzki C. (2015). Arginine deprivation by arginine deiminase of *Streptococcus pyogenes* controls primary glioblastoma growth in vitro and in vivo. Cancer Biol. Ther..

[bib22] Fletcher M., Ramirez M.E., Sierra R.A., Raber P., Thevenot P., Al-Khami A.A., Sanchez-Pino D., Hernandez C., Wyczechowska D.D., Ochoa A.C., Rodriguez P.C. (2015). l-Arginine depletion blunts antitumor T-cell responses by inducing myeloid-derived suppressor cells. Cancer Res..

[bib23] Frauwirth K.A., Riley J.L., Harris M.H., Parry R.V., Rathmell J.C., Plas D.R., Elstrom R.L., June C.H., Thompson C.B. (2002). The CD28 signaling pathway regulates glucose metabolism. Immunity.

[bib24] Friberg M., Jennings R., Alsarraj M., Dessureault S., Cantor A., Extermann M., Mellor A.L., Munn D.H., Antonia S.J. (2002). Indoleamine 2,3-dioxygenase contributes to tumor cell evasion of T cell-mediated rejection. Int. J. Cancer.

[bib25] Fultang L., Vardon A., De Santo C., Mussai F. (2016). Molecular basis and current strategies of therapeutic arginine depletion for cancer. Int. J. Cancer.

[bib26] Fultang L., Booth S., Yogev O., Martins da Costa B., Tubb V., Panetti S., Stavrou V., Scarpa U., Jankevics A., Lloyd G. (2020). Metabolic engineering against the arginine microenvironment enhances CAR-T cell proliferation and therapeutic activity. Blood.

[bib27] Gajewski T.F., Schreiber H., Fu Y.X. (2013). Innate and adaptive immune cells in the tumor microenvironment. Nat. Immunol..

[bib28] Galon J., Costes A., Sanchez-Cabo F., Kirilovsky A., Mlecnik B., Lagorce-Pagès C., Tosolini M., Camus M., Berger A., Wind P. (2006). Type, density, and location of immune cells within human colorectal tumors predict clinical outcome. Science.

[bib29] Geiger R., Rieckmann J.C., Wolf T., Basso C., Feng Y., Fuhrer T., Kogadeeva M., Picotti P., Meissner F., Mann M. (2016). l-Arginine modulates t cell metabolism and enhances survival and anti-tumor activity. Cell.

[bib30] Godfrey L., Crump N.T., Thorne R., Lau I.J., Repapi E., Dimou D., Smith A.L., Harman J.R., Telenius J.M., Oudelaar A.M. (2019). DOT1L inhibition reveals a distinct subset of enhancers dependent on H3K79 methylation. Nat. Commun..

[bib31] Grant C.E., Bailey T.L., Noble W.S. (2011). FIMO: Scanning for occurrences of a given motif. Bioinformatics.

[bib32] Gray S.M., Amezquita R.A., Guan T., Kleinstein S.H., Kaech S.M. (2017). Polycomb repressive complex 2-mediated chromatin repression guides effector CD8^+^ T cell terminal differentiation and loss of multipotency. Immunity.

[bib33] Gubser P.M., Bantug G.R., Razik L., Fischer M., Dimeloe S., Hoenger G., Durovic B., Jauch A., Hess C. (2013). Rapid effector function of memory CD8^+^ T cells requires an immediate-early glycolytic switch. Nat. Immunol..

[bib34] Hadrup S., Donia M., Thor Straten P. (2013). Effector CD4 and CD8 T cells and their role in the tumor microenvironment. Cancer Microenviron..

[bib35] Haines R.J., Pendleton L.C., Eichler D.C. (2011). Argininosuccinate synthase: At the center of arginine metabolism. Int. J. Biochem. Mol. Biol..

[bib36] Hamid O., Robert C., Daud A., Hodi F.S., Hwu W.J., Kefford R., Wolchok J.D., Hersey P., Joseph R.W., Weber J.S. (2013). Safety and tumor responses with lambrolizumab (anti-PD-1) in melanoma. N. Engl. J. Med..

[bib37] Hanahan D., Weinberg R.A. (2011). Hallmarks of cancer: The next generation. Cell.

[bib38] Harding H.P., Novoa I., Zhang Y., Zeng H., Wek R., Schapira M., Ron D. (2000). Regulated translation initiation controls stress-induced gene expression in mammalian cells. Mol. Cell.

[bib39] Harding H.P., Zhang Y., Zeng H., Novoa I., Lu P.D., Calfon M., Sadri N., Yun C., Popko B., Paules R. (2003). An integrated stress response regulates amino acid metabolism and resistance to oxidative stress. Mol. Cell.

[bib40] Heinz S., Benner C., Spann N., Bertolino E., Lin Y.C., Laslo P., Cheng J.X., Murre C., Singh H., Glass C.K. (2010). Simple combinations of lineage-determining transcription factors prime *cis*-regulatory elements required for macrophage and B cell identities. Mol. Cell.

[bib41] Henriet E., Abou Hammoud A., Dupuy J.W., Dartigues B., Ezzoukry Z., Dugot-Senant N., Leste-Lasserre T., Pallares-Lupon N., Nikolski M., Le Bail B. (2017). Argininosuccinate synthase 1 (ASS1): A marker of unclassified hepatocellular adenoma and high bleeding risk. Hepatology.

[bib42] Hodi F.S., O’Day S.J., McDermott D.F., Weber R.W., Sosman J.A., Haanen J.B., Gonzalez R., Robert C., Schadendorf D., Hassel J.C. (2010). Improved survival with ipilimumab in patients with metastatic melanoma. N. Engl. J. Med..

[bib43] Kent W.J., Sugnet C.W., Furey T.S., Roskin K.M., Pringle T.H., Zahler A.M., Haussler D. (2002). The human genome browser at UCSC. Genome Res..

[bib44] Kerry J., Godfrey L., Repapi E., Tapia M., Blackledge N.P., Ma H., Ballabio E., O’Byrne S., Ponthan F., Heidenreich O. (2017). MLL-AF4 spreading identifies binding sites that are distinct from super-enhancers and that govern sensitivity to DOT1L inhibition in leukemia. Cell Rep..

[bib45] Khan A., Fornes O., Stigliani A., Gheorghe M., Castro-Mondragon J.A., van der Lee R., Bessy A., Chèneby J., Kulkarni S.R., Tan G. (2018). JASPAR 2018: Update of the open-access database of transcription factor binding profiles and its web framework. Nucleic Acids Res..

[bib46] Kim D.K., Kanai Y., Choi H.W., Tangtrongsup S., Chairoungdua A., Babu E., Tachampa K., Anzai N., Iribe Y., Endou H. (2002). Characterization of the system L amino acid transporter in T24 human bladder carcinoma cells. Biochim. Biophys. Acta.

[bib47] Kim R., Emi M., Tanabe K. (2007). Cancer immunoediting from immune surveillance to immune escape. Immunology.

[bib48] Kobayashi E., Masuda M., Nakayama R., Ichikawa H., Satow R., Shitashige M., Honda K., Yamaguchi U., Shoji A., Tochigi N. (2010). Reduced argininosuccinate synthetase is a predictive biomarker for the development of pulmonary metastasis in patients with osteosarcoma. Mol. Cancer Ther..

[bib113] Kuznetsova T., Wang S.Y., Rao N.A., Mandoli A., Martens J.H., Rother N., Aartse A., Groh L., Janssen-Megens E.M., Li G. (2015). Glucocorticoid receptor and nuclear factor kappa-b affect three-dimensional chromatin organization. Genome Biol.

[bib49] Lange S.M., McKell M.C., Schmidt S.M., Hossfeld A.P., Chaturvedi V., Kinder J.M., McAlees J.W., Lewkowich I.P., Way S.S., Turner J., Qualls J.E. (2017). l-Citrulline metabolism in mice augments CD4^+^ T cell proliferation and cytokine production *in vitro*, and accumulation in the mycobacteria-infected lung. Front. Immunol..

[bib50] Langmead B., Trapnell C., Pop M., Salzberg S.L. (2009). Ultrafast and memory-efficient alignment of short DNA sequences to the human genome. Genome Biol..

[bib51] Li H., Handsaker B., Wysoker A., Fennell T., Ruan J., Homer N., Marth G., Abecasis G., Durbin R., 1000 Genome Project Data Processing Subgroup (2009). The Sequence Alignment/Map format and SAMtools. Bioinformatics.

[bib52] Liu Q., Stewart J., Wang H., Rashid A., Zhao J., Katz M.H., Lee J.E., Fleming J.B., Maitra A., Wolff R.A. (2017). Reduced expression of argininosuccinate synthetase 1 has a negative prognostic impact in patients with pancreatic ductal adenocarcinoma. PLoS ONE.

[bib53] Lu P.D., Harding H.P., Ron D. (2004). Translation reinitiation at alternative open reading frames regulates gene expression in an integrated stress response. J. Cell Biol..

[bib54] Manna S., Kim J.K., Baugé C., Cam M., Zhao Y., Shetty J., Vacchio M.S., Castro E., Tran B., Tessarollo L., Bosselut R. (2015). Histone H3 Lysine 27 demethylases Jmjd3 and Utx are required for T-cell differentiation. Nat. Commun..

[bib55] Martí i Líndez A.A., Dunand-Sauthier I., Conti M., Gobet F., Núñez N., Hannich J.T., Riezman H., Geiger R., Piersigilli A., Hahn K. (2019). Mitochondrial arginase-2 is a cell-autonomous regulator of CD8^+^ T cell function and antitumor efficacy. JCI Insight.

[bib56] Mellor A.L., Munn D.H. (2003). Tryptophan catabolism and regulation of adaptive immunity. J. Immunol..

[bib57] Miraki-Moud F., Ghazaly E., Ariza-McNaughton L., Hodby K.A., Clear A., Anjos-Afonso F., Liapis K., Grantham M., Sohrabi F., Cavenagh J. (2015). Arginine deprivation using pegylated arginine deiminase has activity against primary acute myeloid leukemia cells in vivo. Blood.

[bib58] Miret J.J., Kirschmeier P., Koyama S., Zhu M., Li Y.Y., Naito Y., Wu M., Malladi V.S., Huang W., Walker W. (2019). Suppression of myeloid cell arginase activity leads to therapeutic response in a NSCLC mouse model by activating anti-tumor immunity. J. Immunother. Cancer.

[bib59] Munder M., Schneider H., Luckner C., Giese T., Langhans C.D., Fuentes J.M., Kropf P., Mueller I., Kolb A., Modolell M., Ho A.D. (2006). Suppression of T-cell functions by human granulocyte arginase. Blood.

[bib60] Mussai F., De Santo C., Abu-Dayyeh I., Booth S., Quek L., McEwen-Smith R.M., Qureshi A., Dazzi F., Vyas P., Cerundolo V. (2013). Acute myeloid leukemia creates an arginase-dependent immunosuppressive microenvironment. Blood.

[bib61] Mussai F., Egan S., Higginbotham-Jones J., Perry T., Beggs A., Odintsova E., Loke J., Pratt G., U K.P., Lo A. (2015). Arginine dependence of acute myeloid leukemia blast proliferation: A novel therapeutic target. Blood.

[bib62] Mussai F., Wheat R., Sarrou E., Booth S., Stavrou V., Fultang L., Perry T., Kearns P., Cheng P., Keeshan K. (2019). Targeting the arginine metabolic brake enhances immunotherapy for leukaemia. Int. J. Cancer.

[bib63] Nicholson L.J., Smith P.R., Hiller L., Szlosarek P.W., Kimberley C., Sehouli J., Koensgen D., Mustea A., Schmid P., Crook T. (2009). Epigenetic silencing of argininosuccinate synthetase confers resistance to platinum-induced cell death but collateral sensitivity to arginine auxotrophy in ovarian cancer. Int. J. Cancer.

[bib64] Ohno T., Kimura Y., Sugimura K., Sagawa A., Jhodo S., Azuma I. (1992). Elevated gene expression of argininosuccinate synthetase in peripheral lymphocytes from systemic lupus erythematosus (SLE) patients. Autoimmunity.

[bib65] Ohshima K., Nojima S., Tahara S., Kurashige M., Hori Y., Hagiwara K., Okuzaki D., Oki S., Wada N., Ikeda J.I. (2017). Argininosuccinate synthase 1-deficiency enhances the cell sensitivity to arginine through decreased DEPTOR expression in endometrial cancer. Sci. Rep..

[bib66] Orlando D.A., Chen M.W., Brown V.E., Solanki S., Choi Y.J., Olson E.R., Fritz C.C., Bradner J.E., Guenther M.G. (2014). Quantitative ChIP-Seq normalization reveals global modulation of the epigenome. Cell Rep..

[bib67] Philip M., Fairchild L., Sun L., Horste E.L., Camara S., Shakiba M., Scott A.C., Viale A., Lauer P., Merghoub T. (2017). Chromatin states define tumour-specific T cell dysfunction and reprogramming. Nature.

[bib68] Powell J.D., Pollizzi K.N., Heikamp E.B., Horton M.R. (2012). Regulation of immune responses by mTOR. Annu. Rev. Immunol..

[bib69] Quek L., Otto G.W., Garnett C., Lhermitte L., Karamitros D., Stoilova B., Lau I.J., Doondeea J., Usukhbayar B., Kennedy A. (2016). Genetically distinct leukemic stem cells in human CD34^−^ acute myeloid leukemia are arrested at a hemopoietic precursor-like stage. J. Exp. Med..

[bib70] Quinlan A.R., Hall I.M. (2010). BEDTools: A flexible suite of utilities for comparing genomic features. Bioinformatics.

[bib71] Rho J.H., Qin S., Wang J.Y., Roehrl M.H. (2008). Proteomic expression analysis of surgical human colorectal cancer tissues: up-regulation of PSB7, PRDX1, and SRP9 and hypoxic adaptation in cancer. J. Proteome Res..

[bib72] Robinson M.D., McCarthy D.J., Smyth G.K. (2010). edgeR: A Bioconductor package for differential expression analysis of digital gene expression data. Bioinformatics.

[bib73] Rodriguez P.C., Zea A.H., Culotta K.S., Zabaleta J., Ochoa J.B., Ochoa A.C. (2002). Regulation of T cell receptor CD3ζ chain expression by L-arginine. J. Biol. Chem..

[bib74] Rodriguez P.C., Quiceno D.G., Zabaleta J., Ortiz B., Zea A.H., Piazuelo M.B., Delgado A., Correa P., Brayer J., Sotomayor E.M. (2004). Arginase I production in the tumor microenvironment by mature myeloid cells inhibits T-cell receptor expression and antigen-specific T-cell responses. Cancer Res..

[bib75] Rodriguez P.C., Quiceno D.G., Ochoa A.C. (2007). l-Arginine availability regulates T-lymphocyte cell-cycle progression. Blood.

[bib76] Ross-Innes C.S., Stark R., Teschendorff A.E., Holmes K.A., Ali H.R., Dunning M.J., Brown G.D., Gojis O., Ellis I.O., Green A.R. (2012). Differential oestrogen receptor binding is associated with clinical outcome in breast cancer. Nature.

[bib77] Russ B.E., Olshanksy M., Smallwood H.S., Li J., Denton A.E., Prier J.E., Stock A.T., Croom H.A., Cullen J.G., Nguyen M.L. (2014). Distinct epigenetic signatures delineate transcriptional programs during virus-specific CD8^+^ T cell differentiation. Immunity.

[bib78] Schindelin J., Arganda-Carreras I., Frise E., Kaynig V., Longair M., Pietzsch T., Preibisch S., Rueden C., Saalfeld S., Schmid B. (2012). Fiji: An open-source platform for biological-image analysis. Nat. Methods.

[bib79] Schreiber R.D., Old L.J., Smyth M.J. (2011). Cancer immunoediting: Integrating immunity’s roles in cancer suppression and promotion. Science.

[bib80] Sen D.R., Kaminski J., Barnitz R.A., Kurachi M., Gerdemann U., Yates K.B., Tsao H.W., Godec J., LaFleur M.W., Brown F.D. (2016). The epigenetic landscape of T cell exhaustion. Science.

[bib81] Shan Y.S., Hsu H.P., Lai M.D., Yen M.C., Chen W.C., Fang J.H., Weng T.Y., Chen Y.L. (2015). Argininosuccinate synthetase 1 suppression and arginine restriction inhibit cell migration in gastric cancer cell lines. Sci. Rep..

[bib82] Shan Y.S., Hsu H.P., Lai M.D., Yen M.C., Luo Y.P., Chen Y.L. (2015). Increased expression of argininosuccinate synthetase protein predicts poor prognosis in human gastric cancer. Oncol. Rep..

[bib83] Spranger S., Gajewski T.F. (2018). Mechanisms of tumor cell-intrinsic immune evasion. Annu. Rev. Cancer Biol..

[bib84] Steggerda S.M., Bennett M.K., Chen J., Emberley E., Huang T., Janes J.R., Li W., MacKinnon A.L., Makkouk A., Marguier G. (2017). Inhibition of arginase by CB-1158 blocks myeloid cell-mediated immune suppression in the tumor microenvironment. J. Immunother. Cancer.

[bib85] Sugimura K., Kimura T., Arakawa H., Ohno T., Wada Y., Kimura Y., Saheki T., Azuma I. (1990). Elevated argininosuccinate synthetase activity in adult T leukemia cell lines. Leuk. Res..

[bib86] Sullivan M.R., Danai L.V., Lewis C.A., Chan S.H., Gui D.Y., Kunchok T., Dennstedt E.A., Vander Heiden M.G., Muir A. (2019). Quantification of microenvironmental metabolites in murine cancers reveals determinants of tumor nutrient availability. eLife.

[bib87] Syed N., Langer J., Janczar K., Singh P., Lo Nigro C., Lattanzio L., Coley H.M., Hatzimichael E., Bomalaski J., Szlosarek P. (2013). Epigenetic status of argininosuccinate synthetase and argininosuccinate lyase modulates autophagy and cell death in glioblastoma. Cell Death Dis..

[bib88] Szlosarek P.W., Grimshaw M.J., Wilbanks G.D., Hagemann T., Wilson J.L., Burke F., Stamp G., Balkwill F.R. (2007). Aberrant regulation of argininosuccinate synthetase by TNF-α in human epithelial ovarian cancer. Int. J. Cancer.

[bib89] Szlosarek P.W., Steele J.P., Nolan L., Gilligan D., Taylor P., Spicer J., Lind M., Mitra S., Shamash J., Phillips M.M. (2017). Arginine deprivation with pegylated arginine deiminase in patients with argininosuccinate synthetase 1-deficient malignant pleural mesothelioma: A randomized clinical trial. JAMA Oncol..

[bib90] Tarasenko T.N., Gomez-Rodriguez J., McGuire P.J. (2015). Impaired T cell function in argininosuccinate synthetase deficiency. J. Leukoc. Biol..

[bib91] Telenius J., Hughes J.R. (2018). NGseqBasic—a single-command UNIX tool for ATAC-seq, DNaseI-seq, Cut-and-Run, and ChIP-seq data mapping, high-resolution visualisation, and quality control. bioRxiv.

[bib92] Thompson E., Cascino K., Ordonez A., Zhou W., Vaghasia A., Hamacher-Brady A., Brady N., Sun I.H., Wang R., Rosenberg A. (2020). Mitochondrial induced T cell apoptosis and aberrant myeloid metabolic programs define distinct immune cell subsets during acute and recovered SARS-CoV-2 infection. medRxiv.

[bib93] Timosenko E., Ghadbane H., Silk J.D., Shepherd D., Gileadi U., Howson L.J., Laynes R., Zhao Q., Strausberg R.L., Olsen L.R. (2016). Nutritional stress induced by tryptophan-degrading enzymes results in ATF4-dependent reprogramming of the amino acid transporter profile in tumor cells. Cancer Res..

[bib94] Timosenko E., Hadjinicolaou A.V., Cerundolo V. (2017). Modulation of cancer-specific immune responses by amino acid degrading enzymes. Immunotherapy.

[bib95] Topalian S.L., Hodi F.S., Brahmer J.R., Gettinger S.N., Smith D.C., McDermott D.F., Powderly J.D., Carvajal R.D., Sosman J.A., Atkins M.B. (2012). Safety, activity, and immune correlates of anti-PD-1 antibody in cancer. N. Engl. J. Med..

[bib96] Tsai C.Y., Chi H.C., Chi L.M., Yang H.Y., Tsai M.M., Lee K.F., Huang H.W., Chou L.F., Cheng A.J., Yang C.W. (2018). Argininosuccinate synthetase 1 contributes to gastric cancer invasion and progression by modulating autophagy. FASEB J..

[bib97] Tyrakis P.A., Palazon A., Macias D., Lee K.L., Phan A.T., Veliça P., You J., Chia G.S., Sim J., Doedens A. (2016). *S*-2-hydroxyglutarate regulates CD8^+^ T-lymphocyte fate. Nature.

[bib98] Uyttenhove C., Pilotte L., Théate I., Stroobant V., Colau D., Parmentier N., Boon T., Van den Eynde B.J. (2003). Evidence for a tumoral immune resistance mechanism based on tryptophan degradation by indoleamine 2,3-dioxygenase. Nat. Med..

[bib99] Vattem K.M., Wek R.C. (2004). Reinitiation involving upstream ORFs regulates *ATF4* mRNA translation in mammalian cells. Proc. Natl. Acad. Sci. USA.

[bib100] Vinson C.R., Hai T., Boyd S.M. (1993). Dimerization specificity of the leucine zipper-containing bZIP motif on DNA binding: Prediction and rational design. Genes Dev..

[bib101] Walsby-Tickle J., Gannon J., Hvinden I., Bardella C., Abboud M.I., Nazeer A., Hauton D., Pires E., Cadoux-Hudson T., Schofield C.J., McCullagh J.S.O. (2020). Anion-exchange chromatography mass spectrometry provides extensive coverage of primary metabolic pathways revealing altered metabolism in IDH1 mutant cells. Commun. Biol..

[bib102] Wang R., Dillon C.P., Shi L.Z., Milasta S., Carter R., Finkelstein D., McCormick L.L., Fitzgerald P., Chi H., Munger J., Green D.R. (2011). The transcription factor Myc controls metabolic reprogramming upon T lymphocyte activation. Immunity.

[bib103] Wellenstein M.D., de Visser K.E. (2018). Cancer-cell-intrinsic mechanisms shaping the tumor immune landscape. Immunity.

[bib104] Werner A., Koschke M., Leuchtner N., Luckner-Minden C., Habermeier A., Rupp J., Heinrich C., Conradi R., Closs E.I., Munder M. (2017). Reconstitution of T cell proliferation under arginine limitation: Activated human T cells take up citrulline *via* L-type amino acid transporter 1 and use it to regenerate arginine after induction of argininosuccinate synthase expression. Front. Immunol..

[bib105] Werner A., Pieh D., Echchannaoui H., Rupp J., Rajalingam K., Theobald M., Closs E.I., Munder M. (2019). Cationic amino acid transporter-1-mediated arginine uptake is essential for chronic lymphocytic leukemia cell proliferation and viability. Front. Oncol..

[bib106] Wilkinson A.C., Ballabio E., Geng H., North P., Tapia M., Kerry J., Biswas D., Roeder R.G., Allis C.D., Melnick A. (2013). *RUNX1* is a key target in t(4;11) leukemias that contributes to gene activation through an AF4-MLL complex interaction. Cell Rep..

[bib107] Wolchok J.D., Kluger H., Callahan M.K., Postow M.A., Rizvi N.A., Lesokhin A.M., Segal N.H., Ariyan C.E., Gordon R.A., Reed K. (2013). Nivolumab plus ipilimumab in advanced melanoma. N. Engl. J. Med..

[bib108] Xu W., Yang H., Liu Y., Yang Y., Wang P., Kim S.H., Ito S., Yang C., Wang P., Xiao M.T. (2011). Oncometabolite 2-hydroxyglutarate is a competitive inhibitor of α-ketoglutarate-dependent dioxygenases. Cancer Cell.

[bib109] Ye J., Kumanova M., Hart L.S., Sloane K., Zhang H., De Panis D.N., Bobrovnikova-Marjon E., Diehl J.A., Ron D., Koumenis C. (2010). The GCN2-ATF4 pathway is critical for tumour cell survival and proliferation in response to nutrient deprivation. EMBO J..

[bib110] Zaytouni T., Tsai P.Y., Hitchcock D.S., DuBois C.D., Freinkman E., Lin L., Morales-Oyarvide V., Lenehan P.J., Wolpin B.M., Mino-Kenudson M. (2017). Critical role for arginase 2 in obesity-associated pancreatic cancer. Nat. Commun..

[bib111] Zea A.H., Rodriguez P.C., Culotta K.S., Hernandez C.P., DeSalvo J., Ochoa J.B., Park H.J., Zabaleta J., Ochoa A.C. (2004). l-Arginine modulates CD3ζ expression and T cell function in activated human T lymphocytes. Cell. Immunol..

[bib112] Zhang Y., Liu T., Meyer C.A., Eeckhoute J., Johnson D.S., Bernstein B.E., Nusbaum C., Myers R.M., Brown M., Li W., Liu X.S. (2008). Model-based analysis of ChIP-Seq (MACS). Genome Biol..

