# Supplementary material for: Chromatin accessibility governs the differential response of cancer and T cells to arginine starvation
Source: Cell Rep. 2021 May 11;35(6):109101. doi: 10.1016/j.celrep.2021.109101 (PMC8131582; doi:10.1016/j.celrep.2021.109101)
Supplement: Document S1. Figures S1–S7 and Table S4 [file mmc1.pdf]

Cell Reports, Volume *35*

Supplemental information

Chromatin accessibility governs the differential response of cancer and T cells to arginine starvation

Nicholas T. Crump, Andreas V. Hadjinicolaou, Meng Xia, John Walsby-Tickle, Uzi Gileadi, Ji-Li Chen, Mashiko Setshedi, Lars R. Olsen, I-Jun Lau, Laura Godfrey, Lynn Quek, Zhanru Yu, Erica Ballabio, Mike B. Barnkob, Giorgio Napolitani, Mariolina Salio, Hashem Koohy, Benedikt M. Kessler, Stephen Taylor, Paresh Vyas, James S.O. McCullagh, Thomas A. Milne, and Vincenzo Cerundolo

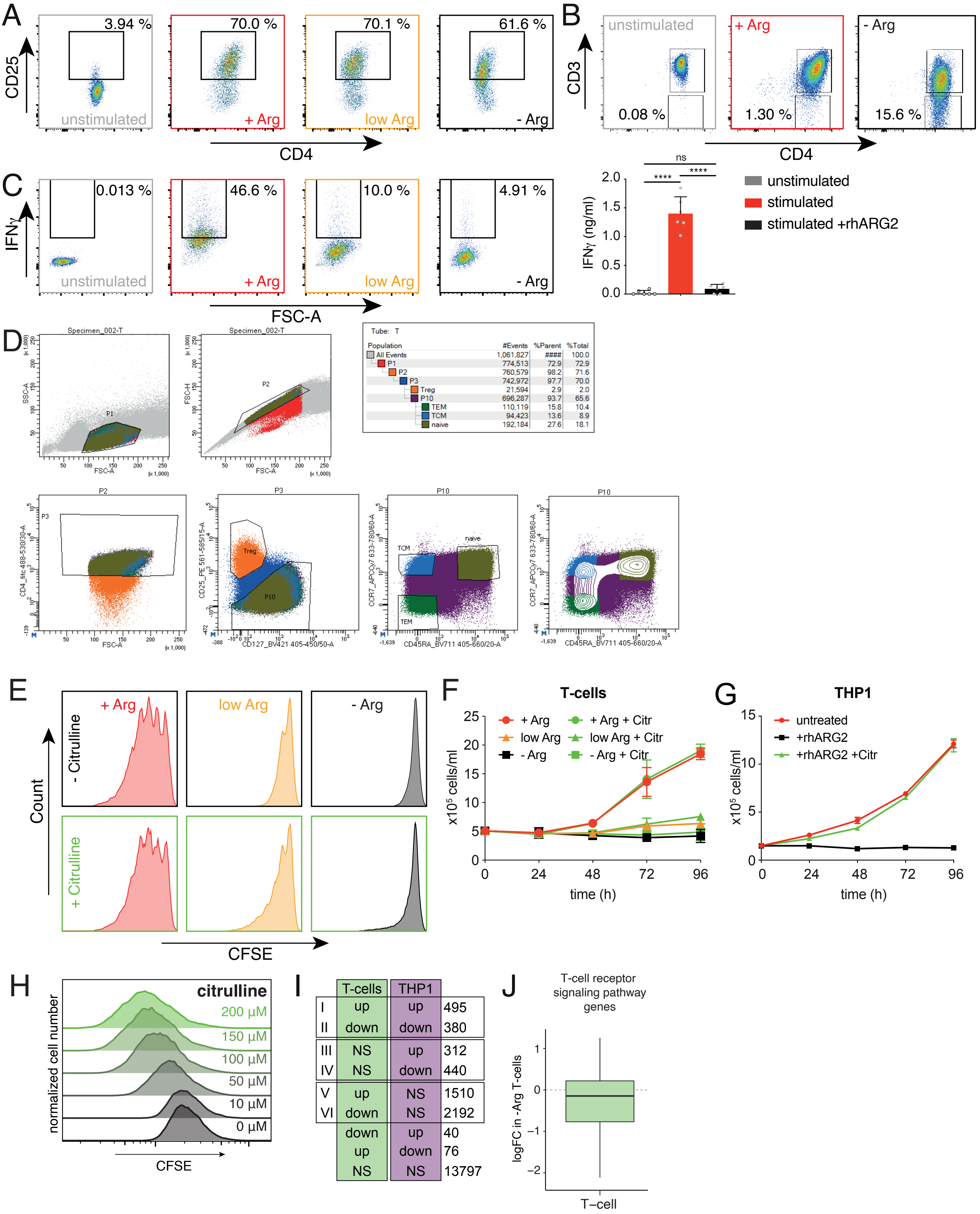
A
3.94 %
70.0 %
70.1 %
61.6 %
CD25
unstimulated
+ Arg
low Arg
- Arg
CD4
B
unstimulated
+ Arg
- Arg
CD3
0.08 %
1.30 %
15.6 %
CD4
C
0.013 %
46.6 %
10.0 %
4.91 %
IFNγ
unstimulated
+ Arg
low Arg
- Arg
FSC-A
ns
****
****
IFNγ (ng/ml)
2.0
1.5
1.0
0.5
0.0
unstimulated
stimulated
stimulated +rhARG2
D
Specimen_002-T
Tube: T
Population #Events %Parent %Total
All Events 1,061,827 #### 100.0
P1 774,513 72.9 72.9
P2 760,579 98.2 71.6
P3 742,972 97.7 70.0
Treg 21,594 2.9 2.0
P10 696,287 93.7 65.6
TEM 110,119 15.8 10.4
TCM 94,423 13.6 8.9
naive 192,184 27.6 18.1
SSC-A
FSC-A
FSC-H
P1
P2
P3
P10
Treg
TCM
TEM
naive
CD4_fitc 488-530/30-A
CD25_PE 561-585/15-A
CD127_BV421 405-450/50-A
CCR7_APCCy7 633-780/60-A
CD45RA_BV711 405-660/20-A
E
+ Arg
low Arg
- Arg
- Citrulline
+ Citrulline
Count
CFSE
F
T-cells
+ Arg
low Arg
- Arg
+ Arg + Citr
low Arg + Citr
- Arg + Citr
x10⁵ cells/ml
time (h)
G
THP1
untreated
+rhARG2
+rhARG2 +Citr
x10⁵ cells/ml
time (h)
H
citrulline
200 µM
150 µM
100 µM
50 µM
10 µM
0 µM
normalized cell number
CFSE
I
T-cells THP1
I up up 495
II down down 380
III NS up 312
IV NS down 440
V up NS 1510
VI down NS 2192
down up 40
up down 76
NS NS 13797
J
T-cell receptor signaling pathway genes
logFC in -Arg T-cells
T−cell

Figure S1. T-cells and THP1 cells show a differential response to arginine starvation, related to Figure 1.

(A) FACS plot showing CD4 and CD25 levels for unstimulated T-cells, or T-cells stimulated in complete medium (+Arg), medium containing 20 µM arginine (low Arg) or in the absence of arginine (-Arg).

(B) Surface expression of CD3 in unstimulated T-cells, or T-cells stimulated in complete medium (+Arg) or in the absence of arginine (-Arg).

(C) IFNγ secretion, measured by FACS (*left*) and ELISA (*right*), from CD4+ T-cells cultured for 96h, either unstimulated or stimulated in complete medium (+ Arg), medium containing 20 µM arginine (low Arg) or lacking arginine (- Arg), or in complete medium supplemented with recombinant human Arginase 2 (rhARG2). Bar chart shows mean ± SD, **** p<0.001, ns: no significant difference (Tukey's multiple comparison test).

(D) Sorting strategy for isolation of CD4+ naïve, central memory T-cells (Tcm) and effector memory T-cells (Tem).

(E) CFSE labeling analysis of stimulated CD4+ T-cells incubated for 96h in complete medium (+Arg), medium containing 20 µM arginine (low Arg) or lacking arginine (-Arg), in the absence (*upper*) or presence (*lower*) of citrulline.

(F) Growth of stimulated CD4+ T-cells, incubated in complete medium (+Arg), medium containing 20 µM arginine (low Arg) or lacking arginine (-Arg), in the absence or presence (+Citr) of citrulline. Cells were counted every 24h for 96h. Data are represented as mean ±SD, n=3.

(G) Growth of THP1 cells incubated in complete medium (untreated) or in equivalent medium treated with recombinant human Arginase 2 with (+rhARG2 +Citr) or without (+rhARG2) the addition of citrulline. Cells were counted every 24h for 96h. Data are represented as mean ±SD, n=4.

(H) CFSE labeling analysis of THP1 cell growth in arginine-free medium, supplemented with the indicated concentrations of citrulline.

(I) Number of upregulated, downregulated and not significantly changed (NS) genes in stimulated T-cells and THP1 cells following 72h incubation in arginine-free medium. See Table S1.

(J) Change in expression (log2(fold-change)) of genes associated with the T-cell receptor signaling KEGG pathway term in stimulated T-cells under arginine starvation. Midline shows median logFC, with upper and lower hinges showing 25th and 75th percentile, respectively. Upper and lower whiskers extend to the largest and smallest datapoints within 1.5 times the interquartile range of either hinge. Gene-specific logFC data are provided in Table S2.

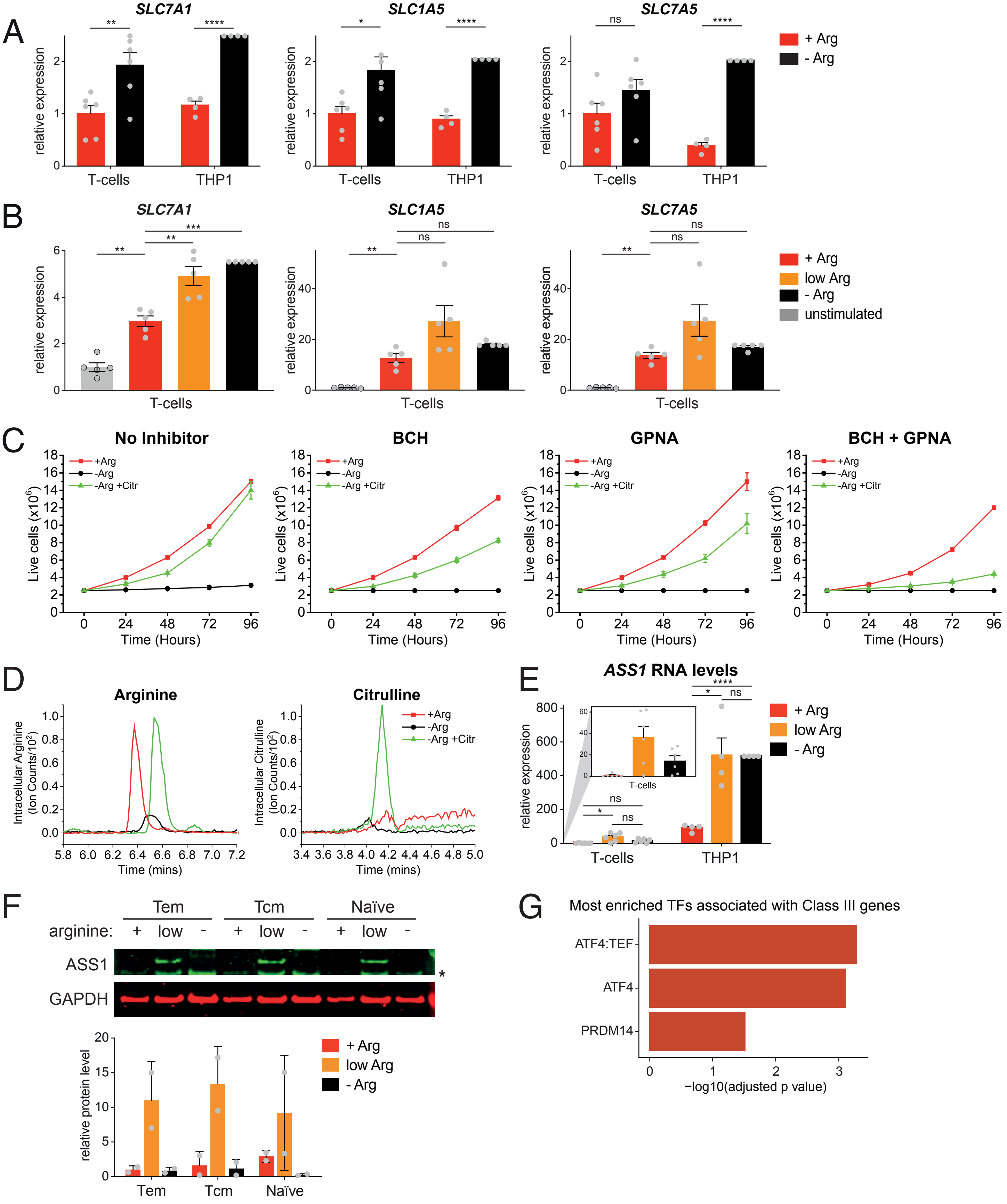

A
SLC7A1
SLC1A5
SLC7A5
relative expression
T-cells
THP1
**
****
*
ns
+ Arg
- Arg
B
***
ns
+ Arg
low Arg
- Arg
unstimulated
C
No Inhibitor
BCH
GPNA
BCH + GPNA
+Arg
-Arg
-Arg +Citr
Live cells (x10^6)
Time (Hours)
D
Arginine
Citrulline
Intracellular Arginine (Ion Counts/10^2)
Intracellular Citrulline (Ion Counts/10^2)
Time (mins)
E
ASS1 RNA levels
F
Tem
Tcm
Naïve
arginine:
+
low
-
ASS1
GAPDH
*
relative protein level
G
Most enriched TFs associated with Class III genes
ATF4:TEF
ATF4
PRDM14
−log10(adjusted p value)

Figure S2. Upregulation of amino acid transporters and arginine biosynthesis in response to arginine starvation, related to Figure 2.

(A) qRT-PCR analysis of transporter gene expression in THP1 and T-cells stimulated in complete medium (+Arg) or in the absence of arginine (-Arg) for 72h. Data are normalized to *YWHAZ*, relative to +Arg T-cells, represented as mean ± SEM, n=4 (THP1), n=6 (T-cell). **** p<0.0001, ** p<0.01, * p<0.05, ns: no significant difference (Dunnett's multiple comparison test).

(B) qRT-PCR analysis of transporter gene expression in T-cells following stimulation in complete medium (+Arg), medium containing 20 µM arginine (low Arg) or lacking arginine (-Arg), or analyzed without stimulation. Data are normalized to *YWHAZ*, relative to unstimulated T-cells, represented as mean ± SEM, n=5. *** p<0.001, ** p<0.01, ns: no significant difference (Dunnett's multiple comparison test).

(C) Growth of THP1 cells treated with the transporter inhibitors GPNA, which targets SLC1A5, and BCH, which targets SLC7A5 (Christensen, 1990; Esslinger et al., 2005; Kim et al., 2002), alone or in combination. Cells were incubated in complete medium (+Arg), medium containing 20 µM arginine (low Arg) or lacking arginine (-Arg), in the absence or presence (+Citr) of citrulline. Cells were counted every 24h for 96h. Data are represented as mean ±SD, n=3.

(D) LC-MS chromatograms showing intracellular arginine and citrulline levels in THP1 cells incubated in complete medium (+Arg) or in arginine-free medium with (-Arg +Citr) or without (-Arg) the addition of citrulline.

(E) qRT-PCR analysis of *ASS1* expression in stimulated T-cells and THP1 cells incubated for 72h in complete medium (+Arg), medium containing 20 µM arginine (low Arg) or lacking arginine (-Arg). *Inset*: expression in T-cells is shown on a smaller scale for clarity. Data are normalized to *YWHAZ*, relative to +Arg T-cells, represented as mean ± SEM, n=4 (THP1), n=6 (T-cell). **** p<0.0001, * p<0.05, ns: no significant difference (Tukey's multiple comparison test).

(F) Representative western blot of ASS1 expression in Tem, Tcm and naïve CD4+ T-cells following 72h incubation in complete medium (+), medium containing 20 µM arginine (low) or lacking arginine (-). Non-specific band is indicated by *. *Lower*: quantification of western blot replicates. Protein levels are normalized to GAPDH, relative to +Arg Tem cells. Data are represented as mean ± SD, n=2.

(G) Significantly enriched transcription factor motifs at the promoters of Class III genes (upregulated in THP1 but not T-cells; see Fig 1D).

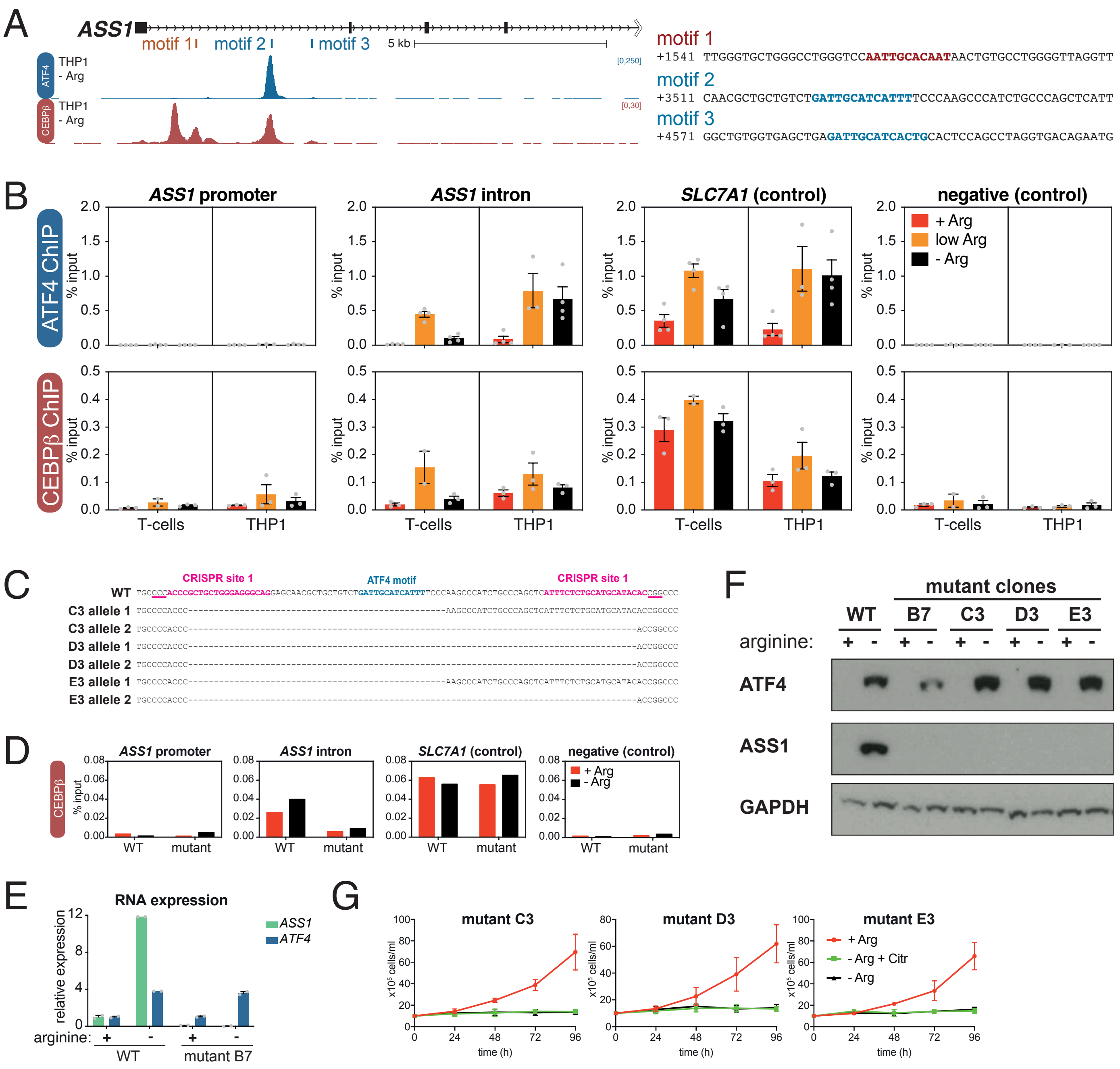
A
ASS1
motif 1
motif 2
motif 3
5 kb
ATF4
THP1 - Arg
CEBPβ
THP1 - Arg
[0,250]
[0,30]
motif 1
+1541 TTGGGTGCTGGGCCTGGGTCCAATTGCACAATAACTGTGCCTGGGGTTAGGTT
motif 2
+3511 CAACGCTGCTGTCTGATTGCATCATTTTCCCAAGCCCATCTGCCCAGCTCATT
motif 3
+4571 GGCTGTGGTGAGCTGAGATTGCATCACTGCACTCCAGCCTAGGTGACAGAATG
B
ASS1 promoter
ASS1 intron
SLC7A1 (control)
negative (control)
ATF4 ChIP
CEBPβ ChIP
% input
+ Arg
low Arg
- Arg
T-cells
THP1
C
CRISPR site 1
ATF4 motif
WT
C3 allele 1
C3 allele 2
D3 allele 1
D3 allele 2
E3 allele 1
E3 allele 2
D
CEBPβ
% input
ASS1 promoter
ASS1 intron
SLC7A1 (control)
negative (control)
+ Arg
- Arg
WT
mutant
E
RNA expression
relative expression
ASS1
ATF4
arginine:
WT
mutant B7
F
mutant clones
WT
B7
C3
D3
E3
arginine:
ATF4
ASS1
GAPDH
G
mutant C3
mutant D3
mutant E3
x10⁵ cells/ml
time (h)
+ Arg
- Arg + Citr
- Arg

Figure S3. ATF4 activates *ASS1* transcription via an intronic enhancer, related to Figure 3.

(A) Location and sequences of ATF4 and CEBPβ motifs within *ASS1* intron 1.

(B) ChIP-qPCR analysis of ATF4 and CEBPβ binding in stimulated T-cells and THP1 cells following 72h incubation in complete medium (+Arg), medium containing 20 μM arginine (low Arg) or lacking arginine (-Arg). Data are represented as mean ± SEM, n=4 (ATF4), n=3 (CEBPβ).

(C) Sequences of the ATF4 binding site in parental (WT) THP1 and three mutant cell lines. Targeted CRISPR sites are indicated in pink, with the PAMs underlined. ATF4 motif is shown in blue.

(D) ChIP-qPCR for CEBPβ in parental (WT) and mutant THP1 cell lines (clone B7; shown in Fig 3C-F), incubated for 72h in complete (+Arg) or arginine-free (-Arg) medium, n=1.

(E) qRT-PCR analysis of *ASS1* and *ATF4* expression in parental (WT) and mutant THP1 cells, incubated for 72h in complete (+) or arginine-free (-) medium. Data are normalized to *GAPDH*, relative to +Arg WT cells, represented as mean ± SD, n=2.

(F) Representative western blot analysis of ASS1 and ATF4 expression in parental (WT) and mutant THP1 cells, incubated for 72h in complete (+) or arginine-free (-) medium. Clone B7 is analyzed in Fig 3C-F. Representative blot of 3 independent replicates.

(G) Growth of mutant THP1 cell lines, incubated in complete medium (+Arg) or in equivalent medium lacking arginine with (-Arg +Citr) or without (-Arg) the addition of citrulline. Cells were counted at 24h intervals for 96h. Data are represented as mean ±SD, n=3. The parental (WT) THP1 growth curve generated with these data is shown in Fig 3F.

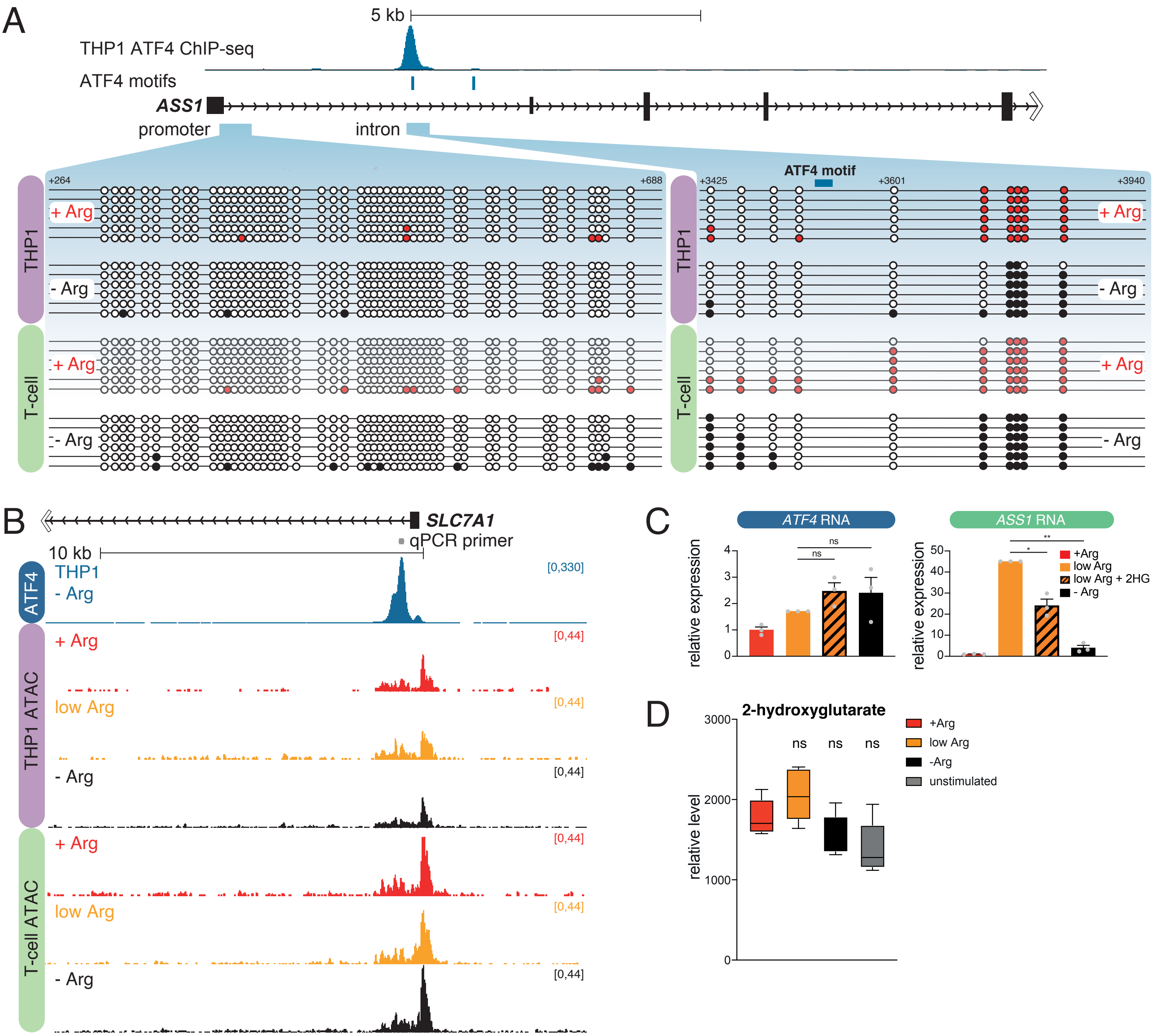

A
5 kb
THP1 ATF4 ChIP-seq
ATF4 motifs
ASS1
promoter
intron
+264
+688
ATF4 motif
+3425
+3601
+3940
THP1
T-cell
+ Arg
- Arg
B
SLC7A1
qPCR primer
10 kb
ATF4
THP1
- Arg
[0,330]
THP1 ATAC
T-cell ATAC
+ Arg
low Arg
- Arg
[0,44]
C
ATF4 RNA
ASS1 RNA
relative expression
ns
*
**
+Arg
low Arg
low Arg + 2HG
- Arg
D
2-hydroxyglutarate
relative level
ns
+Arg
low Arg
-Arg
unstimulated

Figure S4. ***ASS1*** **is repressed in T-cells, related to Figure 4.**

(A) Bisulfite-sequencing analysis of DNA methylation of the *ASS1* promoter and enhancer in THP1 cells or stimulated T-cells cultured in complete medium (+Arg) or arginine-free medium (-Arg). Open circles indicate an unmethylated CpG, closed circles indicated a methylated CpG. Six rows are shown for each condition to indicate the proportion of methylation at each CpG.

(B) ATAC-seq analysis of chromatin accessibility at *SLC7A1* in THP1 and stimulated T-cells following 72h incubation in complete medium (+Arg), medium containing 20 µM arginine (low Arg) or lacking arginine (-Arg).

(C) qRT-PCR analysis of *ASS1* and *ATF4* expression in stimulated T-cells following 72h incubation in complete medium (+Arg), medium containing 20 µM arginine, without (low Arg) or with (low Arg +2HG) supplementation with 500 µM 2-hydroxyglutarate, or lacking arginine (-Arg). Data are normalized to *YWHAZ*, relative to +Arg T-cells, represented as mean ± SEM, n=3. ** $p<0.01$, * $p<0.05$, ns: no significant differences (Dunnett's multiple comparison test).

(D) Levels of 2-hydroxyglutarate detected by LC-MS, taken from T-cells stimulated in complete medium (+Arg), medium containing 20 µM arginine (low Arg) or lacking arginine (-Arg), or CD4+ T-cells analyzed without stimulation. Midline shows median, n=5, with upper and lower hinges showing 25th and 75th percentile, respectively. Upper and lower whiskers extend to the largest and smallest datapoints. ns: no significant differences; comparisons made with +Arg (Dunnett's multiple comparison test).

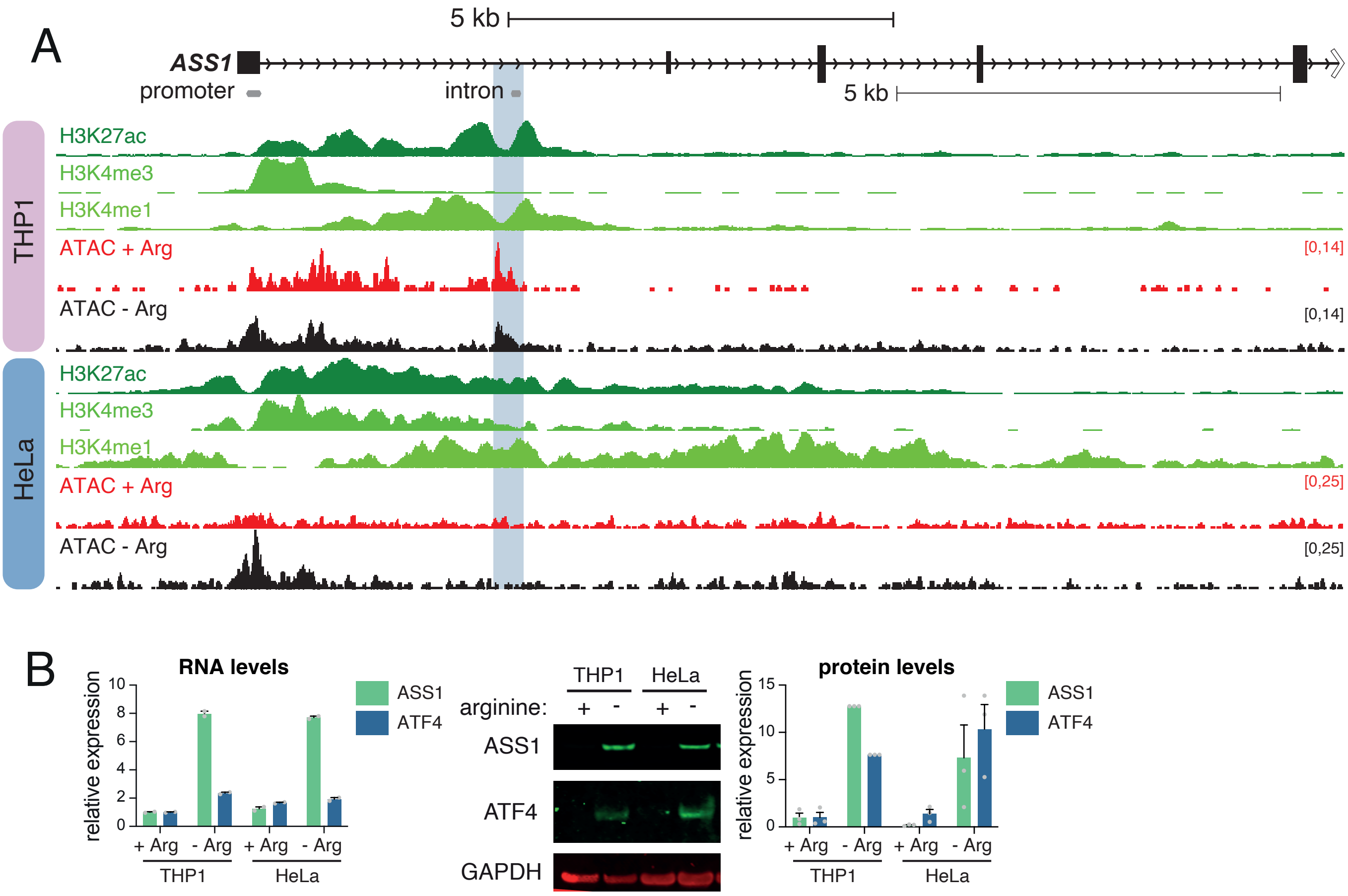

A
5 kb
ASS1
promoter
intron
5 kb
THP1
H3K27ac
H3K4me3
H3K4me1
ATAC + Arg
[0,14]
ATAC - Arg
[0,14]
HeLa
H3K27ac
H3K4me3
H3K4me1
ATAC + Arg
[0,25]
ATAC - Arg
[0,25]
B
RNA levels
relative expression
ASS1
ATF4
+ Arg
- Arg
THP1
HeLa
arginine:
ASS1
ATF4
GAPDH
protein levels

Figure S5. *ASS1* upregulation is a common response to arginine starvation in cancer cells, related to Figure 5.

(A) Publicly available ChIP-seq at *ASS1* for H3K27ac, H3K4me3 and H3K4me1 in THP1 (Godfrey et al., 2019) and HeLa (Kuznetsova et al., 2015) cells in complete medium, and ATAC-seq from THP1 and HeLa cells in complete medium (+Arg) or under arginine starvation (-Arg). The ATF4 binding site is highlighted in blue.

(B) qRT-PCR (*left*) and representative western blot (*right*) analysis of ASS1 and ATF4 expression in THP1 and HeLa cells incubated for 72h in complete (+Arg) or arginine-free (-Arg) medium. Data are normalized to *GAPDH* RNA or protein, relative to THP1 +Arg, presented as mean ±SEM, n=2 (RNA), n=3 (protein).

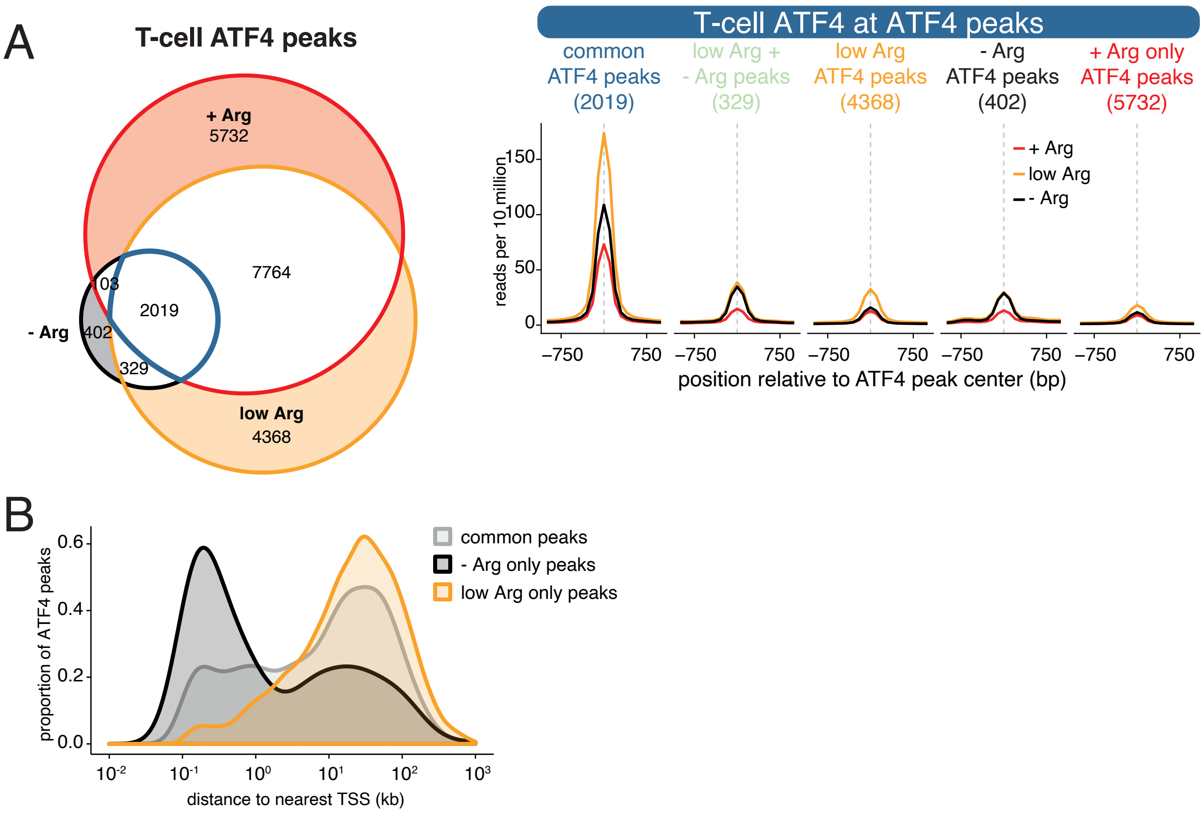
A
T-cell ATF4 peaks
+ Arg
5732
7764
103
2019
- Arg
402
329
low Arg
4368
T-cell ATF4 at ATF4 peaks
common ATF4 peaks (2019)
low Arg + - Arg peaks (329)
low Arg ATF4 peaks (4368)
- Arg ATF4 peaks (402)
+ Arg only ATF4 peaks (5732)
reads per 10 million
+ Arg
low Arg
- Arg
position relative to ATF4 peak center (bp)
B
proportion of ATF4 peaks
common peaks
- Arg only peaks
low Arg only peaks
distance to nearest TSS (kb)

Figure S6. Arginine-starved T-cells show restricted ATF4/CEBPβ binding and reduced chromatin accessibility, related to Figure 6.

(A) *Left*: overlap of ATF4 ChIP-seq peaks identified in stimulated T-cells incubated for 72h in complete medium (+Arg), medium containing 20 µM arginine (low Arg) or lacking arginine (-Arg). *Right*: metaplot analysis of reference-normalized ATF4 ChIP-seq levels in T-cells in complete medium (+Arg; red line), medium containing 20 µM arginine (low Arg; orange line) or lacking arginine (-Arg; black line). The mean level is displayed for ATF4 peaks found: only under complete medium (+Arg only); only under low Arg conditions (low Arg only); only under arginine starvation (-Arg only); present under both low Arg and starvation conditions (low Arg + -Arg); or present under all three conditions (common ATF4 peaks), as illustrated in Venn diagram.

(B) Genomic distribution of T-cell ATF4 peaks. ATF4 peaks found: only under complete medium (+Arg only); only under low Arg conditions (low Arg only); or under both conditions (common; see Fig 6A, *right*) were plotted as a frequency distribution based on the distance to the nearest annotated transcriptional start site (TSS).

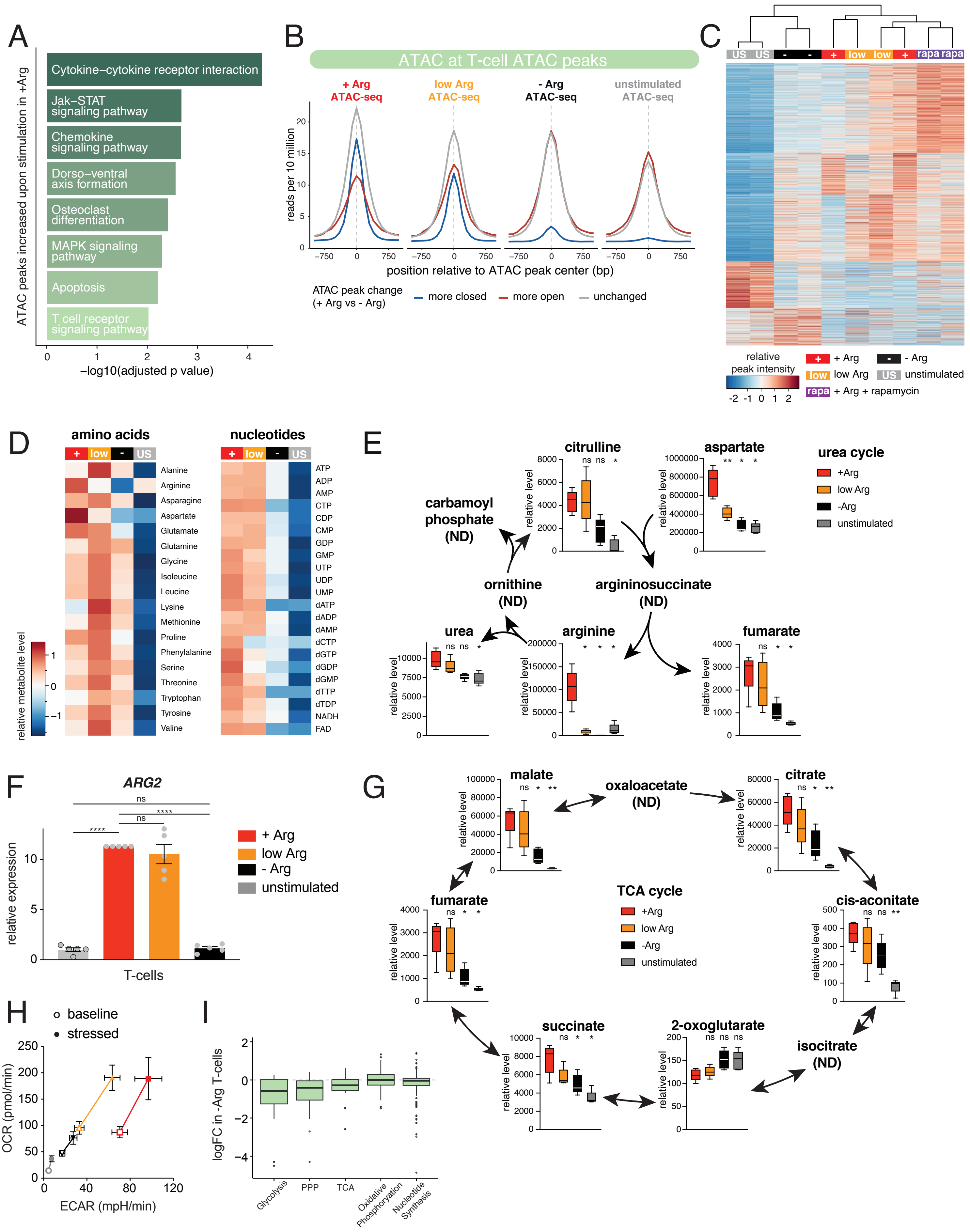

A
Cytokine–cytokine receptor interaction
Jak–STAT signaling pathway
Chemokine signaling pathway
Dorso–ventral axis formation
Osteoclast differentiation
MAPK signaling pathway
Apoptosis
T cell receptor signaling pathway
ATAC peaks increased upon stimulation in +Arg
–log10(adjusted p value)
B
ATAC at T-cell ATAC peaks
+ Arg ATAC-seq
low Arg ATAC-seq
- Arg ATAC-seq
unstimulated ATAC-seq
reads per 10 million
position relative to ATAC peak center (bp)
ATAC peak change (+ Arg vs - Arg)
more closed
more open
unchanged
C
US US - - + low low + rapa rapa
relative peak intensity
+ Arg
- Arg
low Arg
unstimulated
+ Arg + rapamycin
D
amino acids
nucleotides
relative metabolite level
Alanine
Arginine
Asparagine
Aspartate
Glutamate
Glutamine
Glycine
Isoleucine
Leucine
Lysine
Methionine
Proline
Phenylalanine
Serine
Threonine
Tryptophan
Tyrosine
Valine
ATP ADP AMP CTP CDP CMP GDP GMP UTP UDP UMP dATP dADP dAMP dCTP dGTP dGDP dGMP dTTP dTDP NADH FAD
E
citrulline
aspartate
urea cycle
+Arg
low Arg
-Arg
unstimulated
carbamoyl phosphate (ND)
ornithine (ND)
argininosuccinate (ND)
urea
arginine
fumarate
relative level
F
ARG2
relative expression
T-cells
+ Arg
low Arg
- Arg
unstimulated
G
malate
oxaloacetate (ND)
citrate
fumarate
TCA cycle
cis-aconitate
isocitrate (ND)
succinate
2-oxoglutarate
H
baseline
stressed
OCR (pmol/min)
ECAR (mpH/min)
I
logFC in -Arg T-cells
Glycolysis
PPP
TCA
Oxidative Phosphoryation
Nucleotide Synthesis

Figure S7. Arginine starvation disrupts chromatin and metabolic reprogramming associated with T-cell activation, related to Figure 7.

(A) Analysis of KEGG pathway enrichment in the genes associated (closest TSS) with ATAC peaks significantly increased upon T-cell stimulation in complete medium. The eight most significantly-enriched pathway terms are shown.

(B) Metaplot analysis of ATAC-seq levels at ATAC-seq peaks. T-cells were stimulated in complete medium (+Arg), low arginine medium (low Arg) or in the absence of arginine (-Arg), or analyzed without stimulation (unstimulated). Each plot shows the mean ATAC-seq levels in T-cells under the indicated condition at each class of peak. Blue lines: ATAC peaks showing lower accessibility in -Arg T-cells compared to +Arg cells; red lines: ATAC peaks showing greater accessibility in -Arg T-cells; gray lines: ATAC peaks showing no significant difference under arginine starvation.

(C) K-means clustering analysis (k=5) of the 14144 differential ATAC peaks following stimulation in complete medium (see Fig 7A, *left*), using ATAC-seq from T-cells stimulated in complete medium (+), medium containing 20 µM arginine (low) or lacking arginine (-), or complete medium in the presence of 20 nM rapamycin (rapa) for 72h, or CD4+ T-cells analyzed without stimulation (US). Each column is one sample, with each row an ATAC peak. Dendrogram shows the relationships between samples, using a 'friend of friends' clustering algorithm.

(D) Heatmap showing the mean levels of amino acids (*left*) and nucleotides (*right*) detected by LC-MS in T-cells cultured under the indicated conditions.

(E) Levels of urea cycle intermediates detected by LC-MS, taken from T-cells stimulated in complete medium (+Arg), medium containing 20 µM arginine (low Arg) or lacking arginine (-Arg), or CD4+ T-cells analyzed without stimulation. Midline shows median logFC, n=5, with upper and lower hinges showing 25th and 75th percentile, respectively. Upper and lower whiskers extend to the largest and smallest datapoints. ** $p<0.01$, * $p<0.05$, ns: no significant differences; comparisons made with +Arg (Tukey's multiple comparison test). ND, not determined.

(F) qRT-PCR analysis of *ARG2* expression in T-cells following stimulation in complete medium (+Arg), medium containing 20 µM arginine (low Arg) or lacking arginine (-Arg), or analyzed without stimulation. Data are normalized to *GAPDH*, relative to unstimulated T-cells, represented as mean ± SEM, n=5. **** $p<0.0001$, ns: no significant differences (Dunnett's multiple comparison test).

(G) Levels of TCA cycle intermediates detected by LC-MS, taken from T-cells stimulated in complete medium (+Arg), medium containing 20 µM arginine (low Arg) or lacking arginine (-Arg), or CD4+ T-cells analyzed without stimulation. Midline shows median, n=5, with upper and lower hinges showing 25th and 75th percentile, respectively. Upper and lower whiskers extend to the largest and smallest datapoints. ** $p<0.01$, * $p<0.05$, ns: no significant differences; comparisons made with +Arg (Tukey's multiple comparison test). ND, not determined.

(H) Seahorse data from Fig 7H are replotted to show the relative differences in mitochondrial and glycolytic rate under baseline and stressed (after addition of FCCP and oligomycin) conditions.

(I) Change in expression (log2(fold-change)) of genes associated with the indicated metabolic pathways in stimulated T-cells under arginine starvation. Midline shows median, with upper and lower hinges showing 25th and 75th percentile, respectively. Upper and lower whiskers extend to the largest and smallest datapoints within 1.5 times the interquartile range of either hinge. Gene-specific logFC data are provided in Table S2.

Table S4. PCR primers used in this study, related to STAR methods.

Primer	Forward	Reverse	Taqman probe sequence/ID
YWHAZ			Hs03044281_g1
GAPDH			Hs99999905_m1
ATF4			Hs00909569_g1
ASS1			Hs01597989_g1
SLC7A5			Hs01001189_m1
SLC1A5			Hs01056542_m1
SLC7A1			Hs00931450_m1
ARG2			Hs00982833_m1
ASS1 promoter	GAGCGGCTCGGGTTATTT	GCTTCACGACACAGAGGAA	CCGACAAGGAGTCCAGAAAGGACC
ASS1 intron	ATCTGCCCAGCTCATTTCTC	TGCATGTGTGTGATGTGTTTG	
ASS1 intron (used for enhancer deletion study)	ATGCACACAGGCACACAT	GCGTACCTGAGAGTACACAATG	
SLC7A1	CACGCTTACTCACTCGGTGT	GCCTCCACGGAGTCCATTTT	
Negative	GGCTCCTGTAACCAACCACTACC	CCTCTGGGCTGGCTTCATTC	
HOXC8	CAGAAGGGTAGATAGGAGCCTGC	TCAAACAGCGAAGGAGAGGAAG	
ASS1 promoter (bisulfite sequencing)	GTTTTGTGTTTATAATTTGGGATGG	CCCCAAAAAAACTAAACTCCTAATT	
ASS1 enhancer (bisulfite sequencing)	TTTTTGTTTYGGGTTGTAAATGTAGTTTAG	ATAACTAAACAAATATAAACTCTAAAACCTTCAACC	
